# T-pGNN4DTI: Towards better drug-target interactions prediction using Global Self-attentive Pooled Graph Convolutional Networks and protein pre-training Models

**DOI:** 10.1371/journal.pone.0352250

**Published:** 2026-07-13

**Authors:** Yanmei Lin, Boqi Yang, Jianping Liao, Chenjie Du, Hongguo Cai, Yijia Wu, Yuzhong Peng

**Affiliations:** 1 College of Big Data and Software Engineering, Zhejiang Wanli University, Ningbo, China; 2 College of Arts and Sciences, Emory University, Atlanta, United States of America; 3 Guangxi Key Lab of Human-machine Interaction and Intelligent Decision, Nanning Normal University, Nanning, China; 4 Guangdong University of Technology, Zhaoqing, China; Xinjiang Technical Institute of Physics and Chemistry, CHINA

## Abstract

Identification of drug-target interactions (DTI) is an important and challenging task in drug discovery and development. Traditional methods generally require biological experiments, which are costly and time-consuming. Machine learning-based methods can rapidly predict DTI using only computer algorithmic models, allowing researchers to validate only the most promising interactions through biochemical experiments. This holds promise for effectively addressing the current challenges of lengthy development cycles and high costs in new drug development. However, it is difficult for the existing DTI prediction methods to learn complete and effective feature information from the compound and protein. Therefore, this work proposes a DTI prediction method based on the global self-attentive pooled graph neural network and protein pretraining model, called T-pGNN4DTI. On the one hand, T-pGNN4DTI uses a global self-attention pooled graph neural network to learn more meaningful features of the drug molecule by paying more attention to the information features of certain important atomic nodes of the molecular structure and ignoring some weakly relevant node information features. On the other hand, T-pGNN4DTI uses a pre-trained Transformer-based model to capture the semantic relationships of contexts in long sequences of proteins, which can learn more complete feature information. The results of comparing experiments on three benchmark datasets show that the performance of the proposed T-pGNN4DTI model is better than that of the existing DTI prediction methods, effectively improving the DTI prediction. It provides a new way of thinking to help solve the DTI-related problems.

## Introduction

Most drug compounds achieve their therapeutic effects by interacting with specific target molecules (proteins) within the human body to regulate their functions [1]. Therefore, identification of the DTI is one of the most important tasks in the drug discovery process, yet this process using conventional biological experiment-based methods is extremely time-consuming and costly [2–4]. In recent years, advances in artificial intelligence (AI) have enabled machine learning methods to successfully address several complex challenges in drug research and discovery [5–10], driving significant progress in related fields. Consequently, an increasing number of researchers are exploring the application of machine learning methods to address a broader range of complex problems in drug discovery, including DTI prediction. The emergence of AI-based drug-target interaction prediction methods offers an opportunity to alleviate these challenges [3,4,8]. By predicting outcomes, researchers can better explore and understand the mechanisms of drug action, revealing how drugs modulate biological processes to produce therapeutic effects, thereby providing valuable guidance for the design and discovery of new drugs. Accurate and efficient DTI prediction can effectively speed up the virtual screening process of potential compounds [11]. They can minimize unnecessary biological experiments by narrowing the search scope of potential compounds, thus accelerating the development of new drugs [12].

Traditional machine learning methods for DTI prediction can be classified into two categories: similarity-based methods and feature-based methods. Similarity-based methods include Matrix Factorization (MF)-based methods and kernel-based methods. For example, Zheng et al. [12] proposed a Collaborative Matrix Factorization (CMF) based method for DTI prediction; Cichonska et al. [13] proposed a Kronecker product based on compound and protein kernels for DTI prediction. He et al. [14] proposed a classical feature-based DTI prediction method called SimBoost, which defines three types of features for drug, target, and drug-target pairs, respectively, each of which contains multiple hand-created features. These traditional machine learning-based methods, despite achieving good advances in DTI prediction, are still far from the expectations of engineering applications. Numerous studies indicate that deep learning methods outperform traditional machine learning methods in many domains, including drug-target interaction prediction, and hold greater potential for future development [11].

Deep learning-based DTI prediction methods generally include two steps: first, using deep neural networks to learn the features of compounds and proteins from the input raw data; then, the obtained features are used to make classification predictions after completing the trained model. Currently, scholars have developed Convolutional Neural Network (CNN)-based models for feature extraction of compounds and proteins [11,15,16]. However, their difficulty in capturing long-range dependencies results in significant limitations in molecular feature extraction and prevents the representation of potential interactions between atoms at long distances based on the original molecular sequence. To learn more comprehensive drug features, Graph Neural Networks (GNNs) and their variants have also been proposed for extracting features from the 2D structure of compounds [17–19]. For example, Graph Attention Networks (GAT) [20] and Gated Graph Sequence Neural Networks [21] have been used for DTI prediction tasks. Hu et al. [18] proposed a graph neural network-based DTI prediction model, iGRLDTI, which employs an enhanced graph representation learning method to more effectively capture discriminative representations of drugs and targets in the latent feature space, aiming to address the issue of over-smoothing during the simulation process. In recent years, researchers have also employed combinations of different neural networks or attention mechanisms to enhance the accuracy of DTI predictions [22,23]. For example, DeepCDA [24] employs a combination of Long Short-Term Memory (LSTM) and CNN to predict DTI, which represents compounds as text sequences composed of “words” and encodes proteins using word embedding techniques, enabling the LSTM to extract effective features of compounds and proteins from massive unlabeled corpora. DrugBAN [25] utilizes graph convolutional networks (GCNs) and one-dimensional CNNs to extract substructure features from drug molecular graphs and protein sequences, respectively. It then employs a bilinear attention network module to explicitly learn the local interaction relationships between drug-target pairs. AttentionDTA [26] incorporates an attention module after the drug and protein feature extraction stages to emphasize specific features, thereby optimizing DTI prediction outcomes. The application of pre-trained language models (LMs) has become a powerful tool across multiple research domains. BERT (Bidirectional Encoder Representations from Transformers) [27] sparked a paradigm shift in natural language processing tasks, with its influence extending beyond this field. Some pre-trained models for proteins and chemical compounds have been applied in Drug-Drug Interaction (DDI) prediction [28] and DTI prediction studies [29–32], where language models are utilized to generate embedding vectors.

Overall, despite advances in deep learning-based DTI prediction methods, existing approaches still face limitations in feature representation learning and outcome prediction. For instance, when using GCN to learn features of drug compounds, the network treats all atomic nodes as equivalent and assigns them equal weight. This results in certain critical nodes receiving insufficient attention, thereby hindering the identification of key atomic nodes within compound molecular structures. In the process of protein feature extraction, models based on CNN that employ one-hot encoding to extract protein features also exhibit limitations. The primary reason lies in the fact that each protein sequence requires preprocessing before one-hot encoding: longer sequences are truncated, while shorter sequences necessitate padding. This results in the features learned by the subsequent CNN being incomplete. The existing pretrained models generate independent embeddings that disregard neighborhood information. Furthermore, the previous language model-based DTI prediction studies focused solely on comparing different language model variants, lacking comprehensive comparisons with other methods [33].

To tackle the shortcomings of current DTI prediction methods, we studied optimizing feature representation and learning, then proposed a DTI prediction method based on a global self-attention graph convolutional network and a protein pre-training model (T-pGNN4DTI) to empower drug-target interactions prediction.

## Materials and methods

### Benchmark datasets

The dataset employed in this study follows TransformerCPI [34], drawing from three publicly available benchmark datasets widely recognized in the DTA prediction field in recent years [19,35], including the BindingDB, *Human*, and *C.elegans* datasets. Positive samples for the *Human* and *C.elegans* datasets were derived from positive drug-protein interaction (CPI) pairs in DrugBank 4.1 and Matador [34], with high-confidence negative samples generated through the negative CPI screening framework [35]. A more detailed introduction to the three benchmark datasets is given below.

BindingDB is a publicly accessible large-scale database measuring binding affinities, primarily focusing on interactions between proteins considered drug targets and drug-like small molecules [36]. The BindingDB dataset used in this work was obtained from TransformerCPI [34], containing 39747 positive interactions (involving 1696 protein targets and 53253 small molecules) and 31218 negative samples. The *C.elegans* dataset contains extensive information on gene, protein, and compound interactions in the nematode Caenorhabditis elegans, including 4000 positive interactions between 1434 unique compounds and 2,504 unique proteins specific to the *C.elegans* species. The *Human* dataset contains 852 drugs and 1052 proteins, covering 3369 interaction pairs. Table 1 summarizes the statistics of compounds, proteins, and the number of positive and negative samples in each dataset.

**Table 1:** Summary of the datasets.

Dataset	#Compounds	#Protein	#Pairs
BindingDB	53253	1696	39747(+) / 31218(-)
*Human*	852	1052	3369(+) / 3369(-)
*C.elegans*	2504	1434	4000(+) / 4000(-)

## Overview of the model

**Fig 1:**
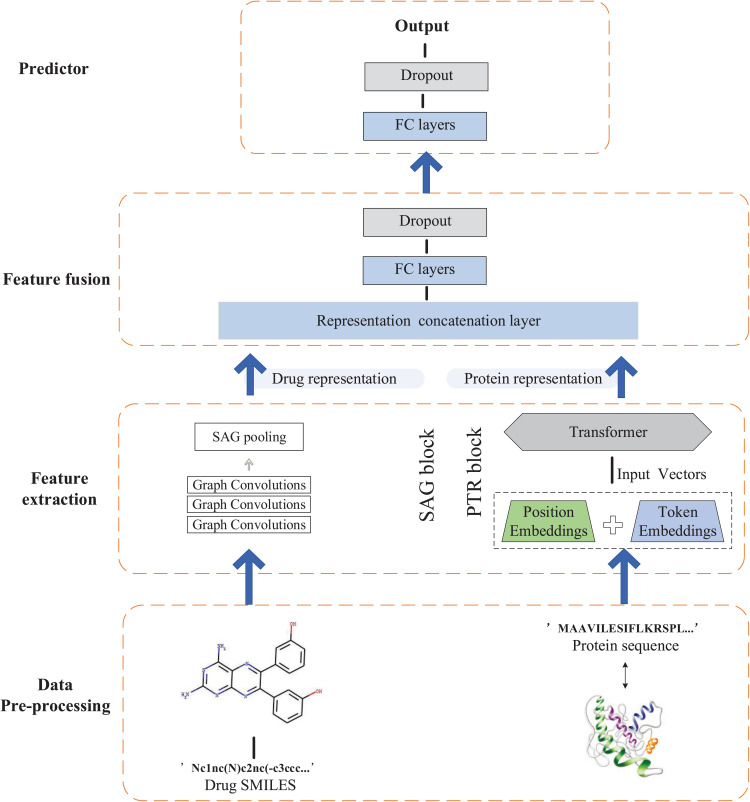
The framework of the proposed T-pGNN4DTI model. T-pGNN4DTI primarily comprises a data preprocessing module (for preprocessing drug data and target protein data, respectively), a feature extraction module (for extracting features from drug data and protein data, respectively), a feature fusion module, and an output module.

The framework of T-pGNN4DTI, shown in Fig 1, consists of four main parts: the data preprocessing module, the feature extraction module, the feature fusion module, and the output module. (1) The data preprocessing module processes the compound data represented by SMILES strings and constructs the corresponding molecular maps of the compounds via the RDKit toolkit. (2) The feature extraction module mainly consists of a global self-attention pooled graph convolutional neural network for learning compound features from molecular maps and a pre-trained model for learning target features from protein sequences. (3) The feature fusion module fuses the features output from the graph convolutional network with the features output from the protein pre-training model for further learning. (4) The output module, consisting of a fully connected layer and a dropout layer, outputs the predicted values of compound-protein pairs.

The technical details of the main modules are as follows:

### Data preprocessing module

**Fig 2:**
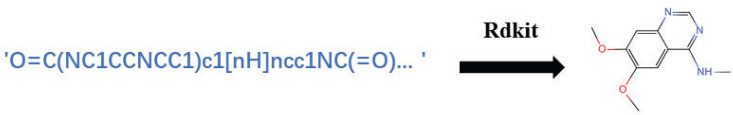
The process diagram of drug SMILES transformation. T-pGNN4DTI utilizes the RDKit to convert drug SMILES into molecular graph data, which contains molecular structural relationships and molecular-related attribute information.

Based on the SMILES of each compound, the tool RDKit was used to construct the corresponding molecular graph reflecting its structural information and inter-atomic interactions, as shown in Fig 2. In the structured molecular graph data, the information of each node can be mapped into a multidimensional feature vector, which mainly describes five pieces of information: the atom symbols, the number of neighboring hydrogens, the number of neighboring atoms, the implied value of the atom, and whether the atom is in an aromatic structure.

T-pGNN4DTI uses a Transformer-based protein pre-training model to learn protein features without processing raw protein data in the data preprocessing module.

### Feature extraction module

#### Feature of the protein extraction module.

With the development of deep learning technology, Transformer-based large-scale pre-trained models (PTMs) have achieved significant success in recent years in various fields, including natural language processing and computer vision. PTMs can efficiently extract knowledge from massive amounts of labeled and unlabeled data, storing this knowledge within a vast parameter system. After fine-tuning for specific downstream tasks, they can support a wide range of downstream applications. On the other hand, protein sequences can also be viewed as a special type of text-based language data, providing favorable conditions for applying language pre-training models to learn protein sequence features.

**Fig 3:**
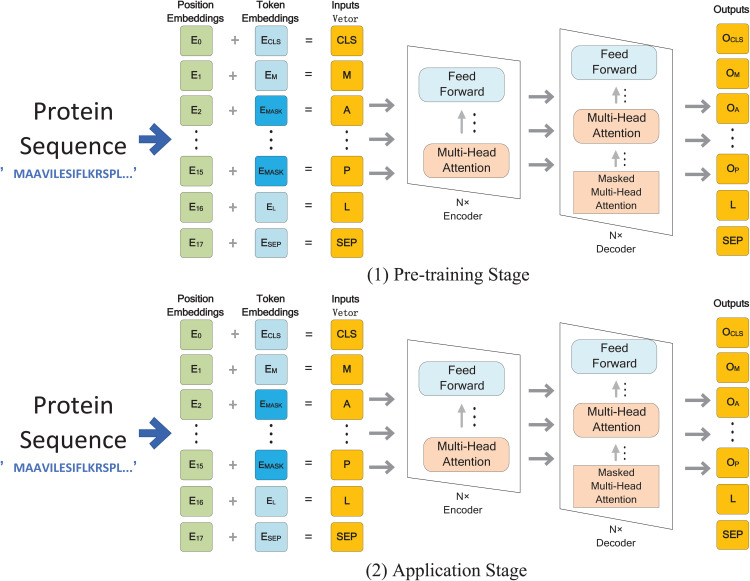
The transformer-based protein pre-training model comprises an encoder block and a decoder module block. Both encoders and decoders process inputs through a sequence of modules alternating between multi-head self-attention layers and feedforward layers.

Inspired by ESM [37], we use a transformer-based protein pre-training model, PTR, for learning protein features in this work. The model is a bidirectional Masked language model, enabling it to observe information (context) near a specific residue or motif and thereby predict that residue or motif. It comprises an encoder module and a decoder module, which are both based on multi-head attention mechanisms and feedforward neural networks, as illustrated in Fig 3. The work process of PTR can be divided into two stages: the pre-training stage and the application stage. The former stage provides the latter stage with a large number of model parameters and contextual relationships within sequences.

In the pre-training phase, the first step is to divide the original protein sequence into several subsequences in terms of characters, starting with the token [CLS] and ending with the token [SEP], $E=\{E_{CLS},E_{1},\cdots ,E_{n},E_{sep}\}$. In the second step, each input vector is obtained by summing the character vectors (Token Embeddings, TE) and character position vectors (Position Embeddings, PE). Where the character vectors are computed using the Word2Vec model [38]. The feed-forward neural network of Word2Vec is used to convert each ID $c_{i}^{*}$, dividing the input sequence into an embedding vector $V_{i}$. The position vector is denoted by PE and is computed using the following equation:


$$\begin{array}{c} \text{PE}(\text{pos},2i)=\sin\left(\frac{\text{pos}}{10000^{2i/d}}\right) \end{array}$$ (1)

$$\begin{array}{c} \text{PE}(\text{pos},2i+1)=\cos\left(\frac{\text{pos}}{10000^{2i/d}}\right) \end{array}$$ (2)

where *pos* denotes the position of the character in the sequence. *d* denotes the dimension of the PE, 2*i* denotes the even dimension, and 2*i* + 1 denotes the odd dimension (2i≤d,2i+1≤d). In the third step, some of the TEs are randomly masked before the vectors are input to the encoder block, and the masked vectors are labeled with MASK. Then the masked characters are predicted by combining the context of the sequence:


$$\begin{array}{c} ℒ(\theta )=-\sum_{i=1}^{M}\log p(m=m_{i}|\theta ),m_{i}\in \{1,2,\ldots ,|C_{p}|\} \end{array}$$ (3)

where *M* is the set of masked characters, $\left|C_{p}\right|$ represents the number of characters in the protein sequence. For each input sequence, a certain proportion of characters is masked, and predictions are then made for the masked portions based on contextual information within the sequence. After extensive training, the model learns to recognize dependencies between masked and unmasked segments within the sequence, thereby enabling more accurate characterization of proteins. In this way, the relationship between contexts that are far apart in the protein sequence can be learned effectively, and features of the protein sequence can be better extracted. Finally, the embedding vector of the protein sequence $IV=\{IV_{1},IV_{2},\cdots ,OV_{L}\}$ is used as input to the PTR model. In the PTR model, *IV* is first fed into the multi-head self-attention layer of the encoder. Within each head of the multi-head self-attention layer, we project the protein features onto query, key, and value vector spaces via projection matrices. The output of each attention head is the result of scaled dot-product attention, as follows,


$$\begin{array}{cc} Attention(Q,K,V) & =aV\\ & =softmax(QK^{T}/{\sqrt{d}}_{k})V \end{array}$$ (4)

where a is the self-attention score matrix, which is an attention weight matrix that follows a probability distribution; $d_{k}$ is the scaling factor (typically set to the dimension of the key vector); *Q*, *K*, and *V* represent the query matrix, key matrix, and value matrix, respectively. They are obtained by multiplying the input features by their corresponding weight matrices. The encoder runs the scaled dot-product attention mechanism demonstrated by Eq. (4) *M* (i.e., the number of heads) times with *M* different queries in parallel and concatenates their outputs. In this way, the self-attention mechanism explicitly constructs pairwise interactions ($QK^{T}$) between all positions in the sequence, enabling the model to represent interactions between residues directly. Additionally, the PTR model adopted a pre-training strategy similar to AlphaFold3.The pre-training corpus is downloaded from RCSB PDB (https://www.rcsb.org, including more than 250000 structures from the PDB archive and 1060000 Computed Structure Models), as well as the protein Swiss-Prot database (https://www.uniprot.org, including about 570000 structures).

In the application phase, similar to the pre-training phase, proteins are first segmented into character-based subsequences. Then, these subsequences are encoded and fed into the model. The huge number of parameters and the contextual correlations in the sequences learned in the pre-training phase are applied when protein features are extracted. Finally, we utilize the feature matrix output from the last layer of the PTR model as the protein representation.

### Drug feature extraction

**Fig 4:**
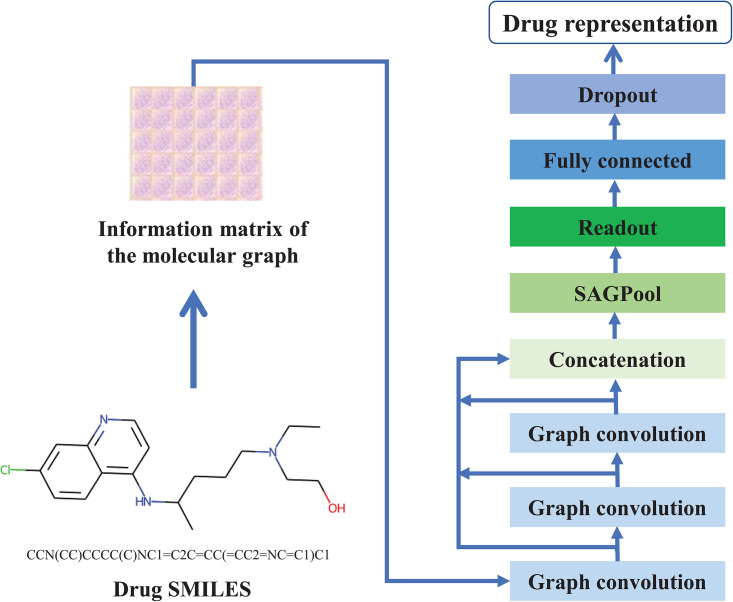
The semantic diagram of the proposed graph convolutional neural network with global self-attention pooling. It describes the pipeline of the proposed graph convolutional neural network.

In recent years, Junhyun Lee et al. [39] proposed Self-Attention Graph Pooling (SAGP), a layer different from the traditional pooling technique in graph neural networks. This method processes input image data by evaluating the relational importance of each node to its neighbors and aggregating based on importance weights, achieving superior performance across numerous image classification tasks. Benefiting from its dynamic weight allocation and hierarchical feature extraction techniques, it demonstrates significant advantages over traditional graph neural networks in noise robustness, structural fault tolerance, and data missing compensation. Inspired by it, to more comprehensively capture the intricate interactions among atoms, between atoms and motifs, and between atoms and motif-functional groups, we propose a Global Self-Attention Pooling-based Graph Convolutional Network (GSAP-GCN). This approach enhances the learning of compound molecular features—including node features and topological structure features—to improve drug-target interaction prediction. The GSAP-GCN framework is shown in Fig 4.

**Fig 5:**
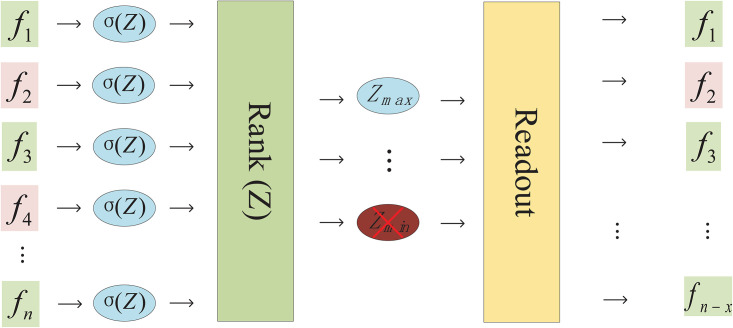
The working mechanism of the global self-attention pooling layer. It illustrates the processing of molecular graph information within the global self-attention pooling layer, where *Z* denotes the attention score.

Given a molecular graph of a chemical compound, *G*=(*V*, *E*). *V* is a set of nodes (i.e., atoms) described by the feature matrix *X* (representing the attributes or features of nodes within the molecular graph). Each node has its own feature vector represented by a *C*-dimensional vector (*C* denotes the number of features of a given node). *E* is the set of edges (i.e., bonds) described by the adjacency matrix *A* (representing the connections between nodes within the molecular graph). The main process of GSAP-GCN to learn the features of this compound is described as follows:

(1) Construct the Node Feature Matrix $X\in R^{N\times C}(N=|V|)$ of the molecular graph of compounds and the adjacency matrix $A\in R^{N\times N}$, which are then inputted to the first graph convolution layer to compute the output node primary feature vectors $H\in R^{N\times d}(d$ denotes the node feature dimension). The computation rule of the graph convolution layer is defined as in Eq (5):
  $$\begin{array}{c} H^{l+1}=\sigma \left({\tilde{D}}^{-\frac{1}{2}}\tilde{A}{\tilde{D}}^{-\frac{1}{2}}H^{l}W^{l}\right) \end{array}$$ (5)
  where $H^{l}$ denotes the feature matrix in layer *l*; $W^{l}$ denotes the weight matrix of layer *l*; $\tilde{A}=A+l_{N}$ is the augmented adjacency matrix with self-connectivity, and $\tilde{D}\in R^{N\times N}$ is the degree matrix of the node. A new feature matrix is first obtained by linearly transforming the mu*l*tiplication by $H^{l}$ and $W^{l}$. Then the weighted sum of the nodes and their neighbors is computed by normalizing the augmented adjacency matrix $\tilde{A}$ (i.e., ${\tilde{D}}^{-\frac{1}{2}}\tilde{A}{\tilde{D}}^{-\frac{1}{2}})$. Finally, a feature matrix in the *l* + 1 layer *H*^(*l*+1)^ is obtained by performing another nonlinear transformation through an activation function σ.
(2) The node feature vectors output from the first graph convolutional layer are input to a second graph convolutional layer for similar computational processing before being input to a third graph convolutional layer, and so on. In particular, a node feature concatenation layer is attached after the last graph convolutional layer, and the output features of each graph convolutional layer are also simultaneously delivered to this concatenation layer.
(3) The concatenation results from the node feature concatenation layer are input into the pooling layer with the self-attention mechanism. The global self-attention pooling layer is capable of filtering nodes, mainly including scoring the importance of nodes and discarding some node information through the masking mechanism. Fig 5 shows the processing of nodes by the global self-attention pooling layer. For ease of description, we denote the input feature matrix of the molecule in the global self-attention pooling layer as $F=[f_{1},f_{2},\ldots ,f_{n}]$. First, to enable simultaneous consideration of node features and graph topological features during molecular information aggregation, our global self-attention pooling layer employs graph convolutions to compute the self-attention scores matrix $Z\in {\mathbb{R}}^{N\times 1}$, as demonstrated in Eq (6).
  $$\begin{array}{c} Z=\sigma \left({\tilde{D}}^{-\frac{1}{2}}\tilde{A}{\tilde{D}}^{-\frac{1}{2}}H^{l}\Theta \right) \end{array}$$ (6)
  where $\Theta \in R^{d\times 1}$ is a learnable convolutional weight matrix; σ(·) is the activation function (the Leaky ReLU function in this work). Alternatively, the softmax function can be used to normalize *Z*, though this comes at the cost of computational efficiency. This calculation method of the self-attention scores matrix differs from the aforementioned PTR network, which calculates self-attention scores using similarity measures that consider only node features, as shown in Eq (4).
  Then, a node pruning pooling method based on the self-attention scores ranking that prioritizes discarding relatively unimportant nodes while retaining only those deemed significant. Specifically, the number of previous [*kN*] nodes is retained based on the order of the self-attention scores. k∈[0,1] represents an adjustable SAG pooling ratio, which is a crucial hyperparameter determining the final model’s prediction performance. The formula for discarding nodes is defined as in Eqs (7) and (8):
  $$\begin{array}{c} idx=top-rank(Z,|kN|) \end{array}$$ (7)
  $$\begin{array}{c} Z_{mask}=Z_{idx} \end{array}$$ (8)
  where, top−rank(U,R) is the function that ranks by *U* and selects the index value of the former *R* nodes; *idx* is the index value; $Z_{mask}\in R^{|mask|\times 1}$ is the attention mask of the feature. Attention mask calculation output process, as shown in Eq (9):
  $$\begin{array}{c} \tilde{X}=X_{idx},X_{out}=\tilde{X}⊙Z_{mask},A_{out}=A_{idx,idx} \end{array}$$ (9)
  where $X_{idx}$ is the feature matrix indexed by node; ⊙ is the broadcasted element-wise product; $A_{idx,idx}$ is the row-wise and column-wise indexed adjacency matrix; $X_{out}\in R^{|mask|\times d}$ and $A_{out}\in R^{\left|mask|\times |mask\right|}$ are the new feature matrix and the corresponding adjacency matrix computed by the attention mask, respectively. In this way, GSAP-GCN can simplify the graph structure while preserving its core structural features and key information, thereby reducing the graph’s size and enhancing computational efficiency.
(4) The retained nodes are then aggregated through the Readout layer to obtain a representation of the graph, which is the feature of the compound. The information of the nodes retained by the global self-attentive pooling layer will be aggregated through the Readout layer. The aggregation is calculated as in Eq (10):
  $$\begin{array}{c} s=\frac{1}{N}\sum_{i=1}^{N}Concat(X_{i},max(X_{i})) \end{array}$$ (10)
  where *N* is the total number of nodes; $X_{i}$ denotes the feature vector of the i−th node and Concat(·) denotes the concatenation operation.
(5) Finally, the aggregated representation vector of the obtained graph is fed into the feed-forward neural network with Dropout operation for further learning and optimization, thereby obtaining more effective drug features $F_{drug}$.

### Feature fusion and output module

In this module, we fuse the compound feature vectors learned from the graph convolutional network with the vectors of protein features output from the pre-trained model for interaction prediction. Specifically, for each compound-protein pair, the drug feature vector $F_{drug}$ learned from the global self-attentive graph neural network and the protein feature vector $F_{protein}$ learned from the PTR network are fused into the feature vector $F_{mixed}=F_{drug}\|F_{protein}$. Finally, the model input $F_{mixed}\in R^{2}$ to the feed-forward neural network with Dropout operation and Softmax output layer, which is used for drug-target interaction prediction, as shown in Eq (11):


$$\begin{array}{c} out=Softmax\left(\sigma \left(W_{f}\cdot F_{mixed}+b_{f}\right)\right) \end{array}$$ (11)

where $W_{f}$ and $b_{f}$ are the weight matrix and bias vector, respectively. *out* is the predicted label of the final output compound-protein pairs.

## Results and discussion

### Experimental Setup

#### Evaluation metrics.

DTI prediction is a classification task, so we employ metrics such as AUC (Area Under the Receiver Operating Characteristic curve), PRC (Area Under the Precision-Recall Curve), Precision, and Recall to evaluate model performance. Precision and Recall are described by the following formulas:


$$\begin{array}{c} Precision=TP/(TP+FP) \end{array}$$ (12)

$$\begin{array}{c} Recall=TP/(TP+FN) \end{array}$$ (13)

where *TP*, *FP* and *FN* are represent the number of true positive, false positive and false negative samples, respectively.

### Baseline models

In this work, the proposed model is compared with state-of-the-art methods, including SAG-DTA [40], TransformerCPI [34], GraphDTA [17],SAG(including SAG_*global*_ and SAG_*hierarchical*_) [40], CPGL [41], AMMVF-DTI [42], MCL-DTI [43], TopoPharmDTI [44], IMAEN [45], MGNDTI [46], CF-DTI [47], and FusionDTI [48] on different datasets.

To verify the advantages of the global self-attention pooling technique used in this work, a T-pGNN4DTI variant model (T-hGNN4DTI) replacing the global self-attention pooling technique with the hierarchical self-attention pooling technique [39] is constructed experimentally to incorporate the SAG_*hierarchical*_ method. SAG_*hierarchical*_ can be divided into three modules, each containing a graph convolution layer and a pooling layer with a self-attention mechanism. The node features obtained from each module are aggregated in the Readout layer. The aggregated node features are summed and then passed through a fully connected layer to obtain a representation of the graph, which is the features of the compound.

In addition, we conducted some t-tests for analyzing the significance of the comparative models’ performance to scientifically determine model superiority.

### Hyperparameter setting

In this work, we followed the dataset partitioning of SAG-DTA [40] that, the BindingDB dataset is partitioned into 80% training set, 10% testing set and 10% validation set, while the other datasets are partitioned into 80% training set and 20% testing set. Hyperparameters used in T-pGNN4DTI and its variant are shown in Table 2, while hyperparameters for state-of-the-art methods remain consistent with the settings in the original literature.

**Table 2:** Hyperparameters used in T-pGNN4DTI and its variant.

Hyperparameters	Setting
Max epoch*	1000
Batch size	512
Optimizer	Adam
Learning rate	0.0005
Dropout rate	0.1
Graph Convolutional layers	3
Pooling ratio	0.8,0.9
FNN hidden layers	3
Early-stopping criteria	monitor=AUC, patience=25, min-delta =0.0001, restore-best-weights = True

The maximum epoch is the maximum number of training epochs in our experimental setup. During actual training, training may terminate early based on the early-stopping criteria.

### Experimental results

In this section, the experimental results for our T-pGNN4DTI and its variant models (T-hGNN4DTI), GCN, FusionDTI, SAG_*global*_, and SAG_*hierarchical*_ are averages obtained from 10 independent runs with different random seeds, respectively. Except for the comparative results of GCN, FusionDTI, SAG_*global*_, and SAG_*hierarchical*_, which were obtained through our repeated experiments, the comparison results for other compared models are cited from the corresponding references.

Tables 3–5 show the performance with P-values of our T-pGNN4DTI model against state-of-the-art methods on the C.elegans, Human, and BindingDB datasets.

**Table 3:** Comparison results on the *C.elegans* dataset.

Method	AUC /P-value	PRC /P-value	Precision /P-value	Recall /P-value
GCN	0.975±0.004 /0.003	0.938±0.005 /0.000	0.921±0.005 /0.000	0.927±0.005 /0.000
GraphDTA [17]	0.974 /0.002	—	0.927 /0.000	0.912 /0.009
CPI-GNN [49]	0.978 /0.011	—	0.938 /0.000	0.929 /0.000
TransformerCPI [34]	0.988 /0.013	—	0.952 /0.000	0.953 /0.001
CPGL [41]	0.990 /0.017	—	0.956 /0.003	0.957 /0.001
IMAEN [45]	0.928 /0.000	—	0.983 /0.013	0.844 /0.000
TopoPharmDTI [44]	0.991 /0.021	—	0.948 /0.001	0.969 /0.002
MCL-DTI [43]	0.992 /0.023	0.994 /0.029	—	0.959 /0.001
MGNDTI [46]	0.991 /0.021	0.991 /0.021	—	—
CF-DTI [47]	0.993 /0.027	0.993 /0.027	0.969 /0.001	0.974 /0.002
SAG_*global*_	0.993±0.003 /0.027	0.992±0.003 /0.023	0.958±0.004 /0.002	0.968±0.004 /0.002
SAG_*hierarchical*_	0.996±0.003 /0.032	0.995±0.003 /0.031	0.981±0.004 /0.005	0.986±0.004 /0.009
FusionDTI	0.996±0.003 /0.032	0.997±0.003 /0.045	*0.991±0.003* /0.028	*0.994±0.004 /0.049*
T-hGNN4DTI	**0.998±0.004 /0.189**	*0.997±0.004 /0.045*	0.989±0.004 /0.012	0.993±0.001 /0.044
T-pGNN4DTI**(Our)**	**0.998±0.003**	**0.998±0.001**	**0.995±0.003**	**0.995±0.001**

Note: The best performances are bolded, and the second performances are in italics. ’—’ means the results were not reported in the references. The comparison results of some baselines are reported without ± SD because the corresponding references did not report the standard deviation for the evaluation metrics.

**Table 4:** Comparison results on the *Human* dataset.

Method	AUC /P-value	PRC /P-value	Precision /P-value	Recall /P-value
GCN	0.956±0.004 /0.001	0.892±0.006 /0.000	0.862±0.008 /0.000	0.928±0.005 /0.004
GraphDTA [17]	0.960 /0.002	—	0.882 /0.000	0.912 /0.001
CPI-GNN [49]	0.970 /0.002	—	0.918 /0.001	0.923 /0.002
DrugVQA [50]	0.964 /0.002	—	0.897 /0.000	0.948 /0.039
TransformerCPI [34]	0.973 /0.003	—	0.916 /0.001	0.925 /0.002
CPGL [41]	0.979 /0.005	—	0.915 /0.001	0.957 /0.016
IMAEN [45]	0.954 /0.001	—	0.930 /0.007	0.857 /0.000
TopoPharmDTI [44]	0.980 /0.005	—	0.920 /0.002	0.949 /0.037
MCL-DTI [43]	0.987 /0.020	*0.989 /0.046*	—	**0.961 /0.009**
MGNDTI [46]	0.986 /0.014	0.982 /0.007	—	0.956 /0.017
AMMVF-DTI [42]	0.986 /0.014	0.976 /0.004	—	0.938 /0.012
CF-DTI [47]	0.987 /0.020	0.983 /0.008	0.939 /0.012	*0.958 /0.013*
SAG_*global*_	0.985±0.009 /0.010	0.986±0.011 /0.014	0.945±0.003 /0.036	0.933±0.005 /0.007
SAG_*hierarchical*_	0.984±0.003 /0.008	0.984±0.003 /0.008	*0.946±0.004 /0.040*	0.931±0.005 /0.006
FusionDTI	0.984±0.002 /0.008	0.984±0.003 /0.008	*0.946±0.005 /0.040*	0.927±0.004 /0.002
T-hGNN4DTI	*0.988±0.002 /0.033*	*0.989±0.002 /0.046*	*0.946±0.004 /0.040*	0.951±0.005 /0.008
T-pGNN4DTI**(Our)**	**0.990±0.002**	**0.990±0.002**	**0.948±0.004**	0.946±0.005

Note: The best performances are bolded, and the second performances are in italics. ’—’ means the results were not reported in the references. The comparison results of some baselines are reported without ± SD because the corresponding references did not report the standard deviation for the evaluation metrics.

**Table 5:** Comparison results on the BindingDB dataset.

Method	AUC /P-value	PRC /P-value	Precision /P-value	Recall /P-value
GCN	0.927±0.005 /0.000	0.913±0.006 /0.000	0.838±0.008 /0.000	0.869±0.007 /0.000
GraphDTA [17]	0.929 /0.000	0.917 /0.000	—	—
CPI-GNN [49]	0.603 /0.000	0.543 /0.000	—	—
TransformerCPI [34]	0.951 /0.007	0.949 /0.004	—	—
IMAEN [45]	0.924 /0.000	0.910 /0.000	—	—
TopoPharmDTI [44]	0.959 /0.011	0.961 /0.011	—	—
MGNDTI [46]	0.952 /0.008	0.935 /0.000	—	0.899 /0.001
CF-DTI [47]	0.965 /0.018	0.955 /0.009	0.896 /0.001	0.910 /0.007
SAG_*global*_	0.954±0.003 /0.011	0.950±0.003 /0.006	0.849±0.006 /0.000	**0.942±0.004 /0.004**
SAG_*hierarchical*_	0.963±0.003 /0.014	0.966±0.003 /0.033	*0.900±0.005 /0.005*	0.882±0.007 /0.000
FusionDTI	**0.972±0.002 /0.101**	*0.972±0.002 /0.044*	0.899±0.003 /0.004	0.922±0.004 /0.044
T-hGNN4DTI	*0.967±0.002 /0.021*	0.967±0.002 /0.020	0.887±0.005 /0.000	*0.923±0.004 /0.190*
T-pGNN4DTI**(Our)**	**0.972±0.002**	**0.973±0.002**	**0.917±0.005**	*0.923±0.003*

Note: The best performances are bolded, and the second performances are in italics. ’—’ means the results were not reported in the references. The comparison results of some baselines are reported without ± SD because the corresponding references did not report the standard deviation for the evaluation metrics.

### Comparisons results

On the *C.elegans* dataset, as shown in Table 3, T-pGNN4DTI outperforms all state-of-the-art models across all performance metrics. It achieves over 0.20% to 2.46% higher AUC values, 0.60% to 6.40% higher PRC values, 1.43% to 8.03% higher Precision values, and 0.91% to 9.10% higher Recall values than the state-of-the-art models, respectively. Notably, the variant T-hGNN4DTI achieved second place, its all performance metrics only slightly lower than T-pGNN4DTI, while also outperforming all state-of-the-art models across all performance metrics on the *C.elegans* dataset. This indicates that the DTI prediction method using the pooled graph neural network to learn drug features and using the protein pretraining model to learn target features may be a better way than existing methods.

On the *Human* dataset, as shown in Table 4, T-pGNN4DTI achieves the best AUC, PRC, and Precision values in comparison with state-of-the-art methods. It achieves over 0.30% to 3.77% higher AUC values, 0.10% to 10.99% higher PRC values, and 0.21% to 9.98% higher Precision values than the state-of-the-art models, respectively. For Recall, MCL-DTI achieved the best value (0.961), which is 1.56% higher than that of our T-pGNN4DTI. Overall, T-pGNN4DTI outperforms state-of-the-art methods in terms of AUC, PRC, and Precision, while only slightly underperforming MCL-DTI in terms of Recall. Notably, the variant T-hGNN4DTI achieved second place in terms of AUC, PRC and Precision, while ranking third in terms of Recall on the *Human* dataset.

On the BindingDB dataset, as shown in Table 5, our T-pGNN4DTI outperforms all state-of-the-art models in terms of AUC, PRC, and Precision. It achieves over 0.93% to 61.19% higher AUC values, 0.52% to 78.82% higher PRC values, and 1.89% to 9.43% higher Precision values than the state-of-the-art models, respectively. For Recall, SAG_*global*_ achieved the best value (0.942), which is 2.06% higher than that of our T-pGNN4DTI. Overall, T-pGNN4DTI outperforms state-of-the-art methods in terms of AUC, PRC, and Precision, while only slightly underperforming SAG_*global*_ in terms of Recall. It achieves a big improvement in terms of AUC and PRC. Notably, the variant T-hGNN4DTI achieved second place in terms of AUC, PRC and Recall on the BindingDB dataset.

### Case and Interpretability for T-pGNN4DTI

**Fig 6:**
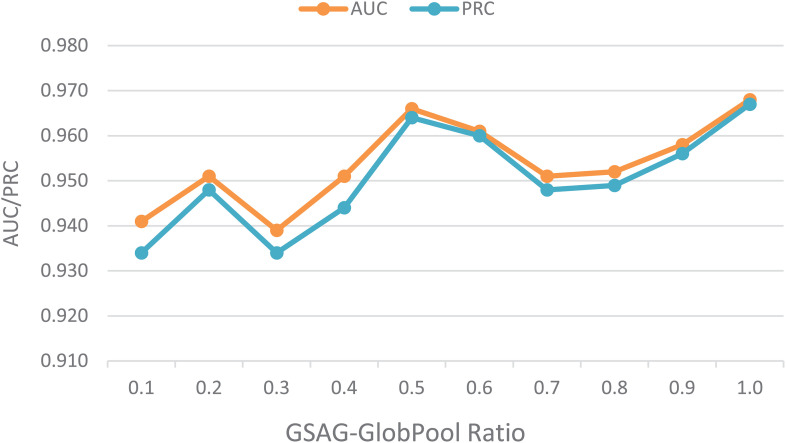
Visualization of Ritonavir feature learning and attention in GSAP-GCN. This picture demonstrates the complete process of GNN learning the molecular features of ritonavir, divided into five subfigures: (A) GSAP-GCN processing flowchart for ritonavir molecules, (B) Pooling process schematic diagram, (C) Attention distribution scatter plot subfigure, (D) Full attention visualization subgraph, and (E) Bar chart for high correlation between GSAP-GCN attention and binding energy.

**Fig 7:**
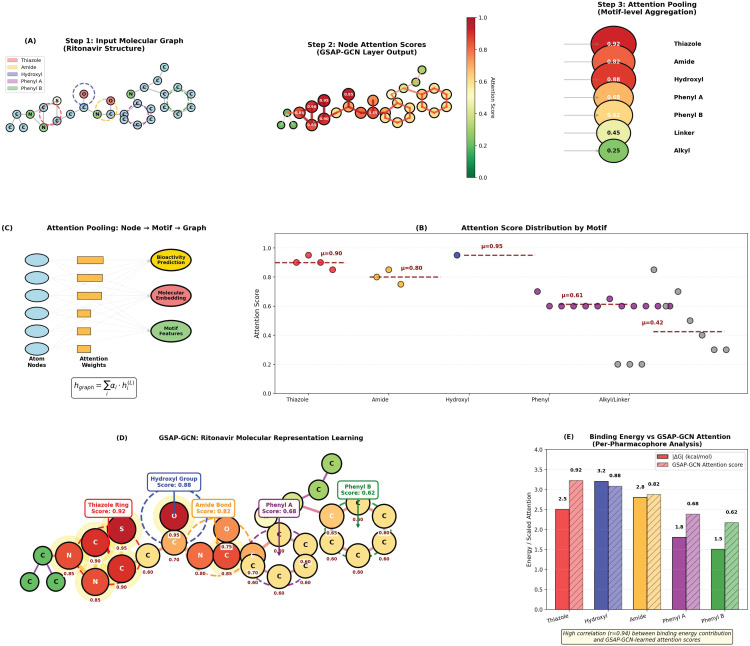
Visualization of the T-pGNN4DTI model learning to model the interaction between Ritonavir and HIV-1 protease. Subfigure (A) describes HIV-1 Protease Structure & Binding Pockets (Ritonavir Binding Sites) in the medical field. Subfigure (B) describes Ritonavir Pharmacophores captured by the attention of GSAP-GCN.

This study employs GSAP-GCN for feature representation learning of compounds, achieving not only excellent DTI prediction performance but also enhancing model interpretability. Specifically, hierarchical pooling dynamically adjusts the contributions of node neighborhoods via attention mechanisms to obtain concise molecular feature vectors that are rich in crucial molecular graph information. By observing the attention coefficients during molecular graph learning, we can measure the relevance of specific node embeddings and neighborhood embeddings to the prediction task. To illustrate this mechanism, we demonstrate and analyze the model’s determination of drug-target interactions between Ritonavir and HIV-1 protease. As shown in Figs 6 and 7

In Fig 6, Subfigure (A) describes GSAP-GCN processing flowchart for ritonavir molecules, which includes three steps: (1) Molecular Input, (2) Node Attention Calculation, and (3) Attention Pooling – Motif-Level Aggregation. Step 1 displays the original structure of ritonavir, with key chemical motifs—including Thiazole, Amide, Hydroxyl, Phenyl A, and Phenyl B—highlighted by dashed lines in different colors. Step 2 displays the atomic-level attention scores output by the GNN layer. Colors range from green (low attention) to red (high attention); node size is proportional to attention scores; edge thickness indicates message-passing weights. Step 3 displays aggregating atomic attention scores into motif importance scores. The thiazole ring achieves the highest score (0.92), indicating its status as a core functional group. This is consistent with pharmaceutical facts in the medical field. It indicates that our model can accurately learn the key motif through which the Ritonavir molecule interacts with HIV-1 protease.

Subfigure (B), a schematic diagram of the pooling process, illustrates the workflow from atom → motif → molecular embedding → bioactivity prediction.

Subfigure (C), an attention distribution scatter plot, displays attention distributions grouped by motif, showing the mean (μ).

Subfigure (D) describes an overall visualization of GSAP-GCN attention, displaying (1) attention scores for atomic nodes, where redder colors indicate higher attention and yellow halos denote key atoms (≥ 0.8); (2) attention scores for chemical bonds, where thickness represents learned message-passing weights; and (3) attention scores for motifs, where dashed ellipses circle important regions and aggregate scores are annotated. Key insights from observing the subfigure (B) include: (1) The thiazole ring (attention score 0.92) is critical for binding to the HIV protease active site; (2) The hydroxyl group (attention score 0.88) serves as a hydrogen bond donor essential for binding; (3) The amide bond (attention score 0.82) plays an important role in binding. This is consistent with pharmaceutical facts in the medical field.

Subfigure (E), the high correlation bar chart, shows the high correlation between GSAP-GCN attention and binding energy. From this subfigure, we observe the following: (1) The correlation coefficient r = 0.94 indicates that the attention scores learned by the GNN highly align with actual binding energy contributions; (2) Despite its small molecular weight, the hydroxyl group receives high attention (0.88) due to the critical role of hydrogen bonding; (3) The thiazole ring achieves the highest attention (0.92) attributable to its large hydrophobic surface area and shape complementarity. This energy-attention comparison validates the physical interpretability of our model.

Overall, Fig 6 demonstrates that GSAP-GCN can automatically identify key pharmacophores through attention mechanisms without requiring manual feature engineering. Furthermore, the finding is consistent with pharmaceutical facts of the interaction between Ritonavir and HIV-1 protease in the medical field, as shown in Fig 7. It is precisely one of the core advantages of T-pGNN4DTI for DTI prediction.

### Influence of the pooling rate in T-pGNN4DTI

The pooling ratio is one of the most important parameters of the global self-attentive pooling layer, which determines the number of nodes retained by the compound molecules in the DTI prediction task and can directly affect the final performance of the T-pGNN4DTI model. To identify the optimal pooling ratio, we evaluated the model performance influence of ten distinct pooling ratios (ranging from 0.1 to 1.0) on the most widely used benchmark dataset, BindingDB.

**Fig 8:**
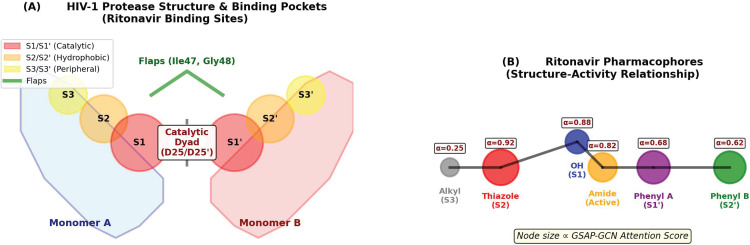
The performance influence of different pooling rates. Different pooling rates resulted in significant differences in both AUC and PRC values of the corresponding model.

Fig 8 illustrates the AUC and PRC values predicted by T-pGNN4DTI when predicting drug-target interactions using different pooling ratios on the BindingDB dataset. It can be observed that both AUC and PRC values show an increasing trend as the pooling ratio increases, with the model achieving optimal performance at a pooling ratio of 0.8. This experimental result reveals that all atoms within the drug molecule have a specific contribution to the drug-protein target interaction. Although assigning weights to the nodes can distinguish the contributions of different atoms and thus improve the performance of the prediction model, those atoms with less attention cannot be completely ignored.

## Results of the ablation experiment

In this section, the ablation experiments were designed and conducted on the BindingDB and *Human* datasets to explore the contribution of the global self-attention pooling technique and the PTR pretrained model to the final prediction results of T-pGNN4DTI. First, the global self-attention pooling technique in T-pGNN4DTI is replaced with the most commonly used global max pooling technique in graph convolutional neural networks and BiLSTM, respectively, while the rest of the model structure remains unchanged. This means replacing the proposed model’s global self-attention pooling graph neural network with a standard graph convolutional neural network and BiLSTM to learn drug features, respectively. The corresponding variant models are named T-GCN and T-BiLSTM, respectively. Second, the PTR model in T-pGNN4DTI is replaced with other protein learning models used for learning protein features, including the one-dimensional convolutional neural network (1-D CNN) [40], ESM-1v [51], and ProteinBERT [52], respectively, while the rest of the model structure remains unchanged. These corresponding variant models are named CNN-pGNN, ESM1v-pGNN, and ProBERT-pGNN, respectively. Table 6 demonstrates the comparative results of the ablation experiments. On the benchmark dataset, T-pGNN4DTI demonstrated significantly superior predictive performance across all four evaluation metrics compared to its variants without global self-attention pooling layers and without protein pre-trained models.

**Table 6:** Ablation experiments and results.

	BindingDB				*Human*			
Model	AUC	PRC	Precision	Recall	AUC	PRC	Precision	Recall
T-BiLSTM	0.955	0.959	0.811	0.878	0.938	0.919	0.804	0.844
T-GCN	0.959	0.958	0.813	0.882	0.959	0.958	0.813	0.882
CNN-pGNN	0.938	0.933	0.812	0.844	0.933	0.941	0.807	0.857
ESM1v-pGNN	0.969	0.968	0.899	0.920	0.979	0.988	0.923	0.942
ProBERT-pGNN	0.968	0.963	0.892	0.914	0.973	0.981	0.927	0.937
T-pGNN4DTI	0.972	0.971	0.917	0.923	0.990	0.990	0.948	0.946

CNN-pGNN replaces the PTR model in T-pGNN4DTI with a one-dimensional convolutional neural network, employing GNNs to learn drug features while utilizing CNNs to learn target features. In contrast, T-pGNN4DTI, T-GCN, and T-BiLSTM employ the PTR model to learn protein features and utilize GNNs to learn drug features. CNN-pGNN underperforms the models T-pGNN4DTI and T-GCN on all datasets across all metrics. T-GCN, which replaces the global self-attention pooling graph neural network in T-pGNN4DTI with a conventional GCN to learn drug features, demonstrates significantly poorer performance than T-pGNN4DTI on all datasets across all metrics. These demonstrate that the global self-attention pooling technique and the PTR pretrained model both have a significant contribution to the final prediction results of T-pGNN4DTI.

As shown in Table 6, all comparative models underperformed T-pGNN4DTI across all datasets and metrics. ESM1v-pGNN, ProBERT-pGNN, and T-BiLSTM all underperformed T-pGNN4DTI across all datasets and metrics. This could be attributed to the excellent synergistic complementarity between the PTR and the global attention pooling graph network with the feed-forward neural network in T-pGNN4DTI, enabling its excellent performance in DTI prediction.

## Discussion

Our experimental results across three benchmark datasets clearly demonstrate that T-pGNN4DTI achieves overall optimal performance, surpassing existing methods in 10 out of 12 evaluations and establishing a new state-of-the-art. This demonstrates that our model’s architecture strategy—based on a global self-attention pooling graph neural network and pre-trained models—is highly effective.

In comparative experiments, T-pGNN4DTI and T-hGNN4DTI demonstrated superior performance across three benchmark datasets, further validating that DTI prediction methods combining GSAP-GCN with the protein PTR model outperform existing approaches. This advantage stems from the ability of GSAP-GCN and protein PTR models to effectively learn local and global feature information of drugs and proteins, respectively. Furthermore, their synergistic complementarity efficiently integrates the feature information of drugs and targets within drug-target pairs, comprehensively capturing key biological features in drug-target interactions.

T-pGNN4DTI outperformed or equaled T-hGNN4DTI in 11 out of 12 evaluations, indicating that the GCN incorporating global self-attention pooling layers in T-pGNN4DTI learns drug features more effectively than the GCN with hierarchical self-attention pooling layers. Furthermore, T-pGNN4DTI and T-hGNN4DTI outperform T-GCN across all 12 evaluations, as shown in Table 6, suggesting that GCNs incorporating self-attention pooling layers are more effective than those without such layers in molecular graph feature learning scenarios.

The result in the ablation experiment, as shown in Table 6, suggests that our model and its variants (replacing the PTR model with ESM-1v[51], and ProteinBERT [52]) leverage pre-trained protein language models to learn protein representations and their features. This approach outperforms methods such as 1-D CNN and BiLSTM, thereby improving DTI prediction accuracy. This finding strongly aligns with the results from FusionDTI [48] and DTI-LM [33], both of which employ protein pre-trained models to learn protein features for DTI prediction.

Overall, T-pGNN4DTI integrates a global self-attention graph-based neural network and a pre-trained model for DTI prediction, which provides a new way of thinking for solving other DTI-related problems.

However, the benchmarking datasets are not ideal due to limitations in data collection methods and experimental conditions. For example, most drugs in the *Human* and BindingDB dataset only occur in one class, potentially introducing bias into feature learning. The relatively small sample size of compound-protein interactions (CPIs) in *Human* and *C.elegans* may limit the training of complex deep learning models. Furthermore, negative samples are generated by algorithms that may introduce undetectable noise [35], necessitating biological experimental corrections by the scientific community.

## Conclusion

In this work, we proposed a DTI prediction method based on a self-attention pooled graph neural network and a pre-trained model, named T-pGNN4DTI. T-pGNN4DTI uses a global self-attention graph convolutional neural network to learn the drug features and uses a pre-trained model based on Transformer to learn the protein features. Experimental comparison results based on three benchmark datasets show that the proposed T-pGNN4DTI model outperforms state-of-the-art methods. The experimental comparison results also demonstrate that using global self-attention pooling graph neural networks and protein pre-trained models to effectively learn drug and target feature information, respectively, is an effective method for improving DTI prediction.

Although the proposed model achieves high predictive performance, its lack of interpretability impedes the understanding of underlying drug–target interaction mechanisms. In addition, it does not fully explore physical constraint information about molecules, which may lead to expertise bias in practical applications and affect its generalization capability. In future research, we will explore methods to enhance model interpretability and incorporate physical constraints into dynamic thermal analysis prediction models to further improve their predictive capabilities.

## Supporting information

S1 File The detailed training strategies of the PTR model. (DOCX)

S2 File Time Complexity and Computational Efficiency. (DOCX)

S3 File Raw datasets. (RAR)
