# Supplementary material for: T-pGNN4DTI: Towards better drug-target interactions prediction using Global Self-attentive Pooled Graph Convolutional Networks and protein pre-training Models
Source: PLoS One. 2026 Jul 13;21(7):e0352250. doi: 10.1371/journal.pone.0352250 (PMC13362149; doi:10.1371/journal.pone.0352250)
Supplement: S1 File — (DOCX) [file pone.0352250.s001.docx]

Supplementary information for

**T-pGNN4DTI: Towards better drug-target interactions prediction using Global Self-attentive Pooled Graph Convolutional Networks and protein pre-training Models**

Yanmei Lin^1‡^, Boqi Yang^2‡^, Jianping Liao^3^, Chenjie Du^1^, Hongguo Cai^3^, Yijia Wu^4*^, Yuzhong Peng ^1*^

**^1^College of Big Data and Software Engineering, Zhejiang Wanli University, 315000 Ningbo, China**

**^2^College of Arts and Sciences, Emory University, 30322 Atlanta, United States**

**^3^Guangxi Key Lab of Human-machine Interaction and Intelligent Decision, Nanning Normal University, 530001 Nanning, China**

**^4^Guangdong University of Technology, 526100 Zhaoqing, China**

**S1.1 The detailed training strategies of the PTR model**

The PTR model adopted a pre-training strategy similar to AlphaFold3. Training comprises four stages: First, the model is trained with random initialization using a sequence cropping size of 384. Subsequently, fine-tuning is performed with a cropping size of 640 based on the weights from the first stage, followed by secondary fine-tuning with a cropping size of 768. During the first fine-tuning stage, the smooth LLDT loss is disabled, and weights from the disordered protein PDB distillation set are reduced. However, unmasked diffusion loss for non-protein chains within this distillation set is now enabled. In the second fine-tuning stage, the transcription factor distillation set is additionally enabled, and weights from the disordered protein PDB distillation set are restored to their original levels. Protein residues from the DNA motif distillation set were considered unresolved if their experimentally resolved probability prediction was below 0.9. In the third (final) fine-tuning stage, we removed all structure-based loss terms (diffusion and distribution map) during training and increased the maximum chain length to 50. All other settings remained consistent with the previous fine-tuning stage.
