# Supplementary material for: T-pGNN4DTI: Towards better drug-target interactions prediction using Global Self-attentive Pooled Graph Convolutional Networks and protein pre-training Models
Source: PLoS One. 2026 Jul 13;21(7):e0352250. doi: 10.1371/journal.pone.0352250 (PMC13362149; doi:10.1371/journal.pone.0352250)
Supplement: S2 File — (DOCX) [file pone.0352250.s002.docx]

Supplementary information for

**T-pGNN4DTI: Towards better drug-target interactions prediction using Global Self-attentive Pooled Graph Convolutional Networks and protein pre-training Models**

Yanmei Lin^1‡^, Boqi Yang^2‡^, Jianping Liao^3^, Chenjie Du^1^, Hongguo Cai^3^, Yijia Wu^4*^, Yuzhong Peng ^1*^

**^1^College of Big Data and Software Engineering, Zhejiang Wanli University, 315000 Ningbo, China**

**^2^College of Arts and Sciences, Emory University, 30322 Atlanta, United States**

**^3^Guangxi Key Lab of Human-machine Interaction and Intelligent Decision, Nanning Normal University, 530001 Nanning, China**

**^4^Guangdong University of Technology, 526100 Zhaoqing, China**

**S2** **Time Complexity and Computational Efficiency**

To estimate the time complexity of our framework, we can first decompose the model and independently assess the time complexity of its three core components as follows:

**(1) Time Complexity of the** **PTR Model:**

Our PTR model is based on the Transformer architecture, where the following factors primarily determine its pre-training time complexity:

Sequence Length (𝑇): The number of amino acids in the protein sequence.

Hidden layer dimension (𝐷): Size of the feature vector.

Number of layers (𝐿): Number of layers in the Transformer encoder.

The core time complexity formula is 𝑂(𝐿⋅𝑇²⋅𝐷)

This complexity stems from the self-attention mechanism requiring computation of all amino acid pair relationships within the sequence (the 𝑇² term).

**(2) Time Complexity of GSAP-GCN:**

GSAP-GCN's time complexity is primarily determined by: the number of network layers *L_g_*, the number of edges 𝐸 in the molecular graph, the number of nodes (atoms) 𝑉 in the molecular graph, and the feature dimension *F* of the node features.

Therefore, the core time complexity formula for GSAP-GCN is *O*(*L_g_⋅*(*E⋅F²*) *+ N⋅F*)

**(3) Time Complexity of FNN:**

The time complexity of full connected FNN is primarily determined by the following factors: the dimension of protein features *D*, the dimension of compound features *F*, the number of hidden layers *L_f_* in the FFN, and the size of the output features *N_f_*.

The core time complexity formula is: *O(N_f_⋅*(*D+F*)*+*( *L_f_* *−1*)*⋅O*(*N_f_*))

To facilitate the construction of deep feedforward neural networks and complexity analysis, the input dimension of the intermediate hidden layer is typically set equal to its output dimension. That is, *N*= *D+F*. Therefore, the core time complexity can be simplified as: *O*(*L_f_⋅*(*D+F*))

**(4) Total Time Complexity and Computational Efficiency of T-pGNN4DTI:**

Since protein feature extraction and compound feature extraction are performed in parallel, the total time complexity is the sum of both plus the FFN component, as follows:

*O*(T-pGNN4DTI) = *O*(PTR) + *O*(GSAP-GCN) + *O*(FFN)

= 𝑂(𝐿⋅𝑇²⋅𝐷)+ *O*(*L_g_⋅*(*E⋅F²*) *+ N⋅F*)+ *O*(*L_f_⋅*(*D+F*))

Since T is much larger than N, the time complexity of T-pGNN4DTI primarily depends on the time complexity of the PTR model. In practical DTI prediction applications, T averages around 1000, N averages around 50, and the PTR model accounts for over 99% of the computational overhead. In our experiments, the GPU used was an NVIDIA RTX 4080 Ti, achieving an inference time of approximately 100 milliseconds per drug-target pair sample. Consequently, T-pGNN4DTI can scale for DTI prediction in higher-performance GPU server cluster environments.
