# Supplementary material for: T-pGNN4DTI: Towards better drug-target interactions prediction using Global Self-attentive Pooled Graph Convolutional Networks and protein pre-training Models
Source: PLoS One. 2026 Jul 13;21(7):e0352250. doi: 10.1371/journal.pone.0352250 (PMC13362149; doi:10.1371/journal.pone.0352250)
Supplement: Attachment — Submitted filename: Response to Reviewers20260602.pdf [file pone.0352250.s006.pdf]

Dear reviewers,

We are very grateful to the editor and all reviewers for their hard work in reviewing our paper. In this round of reviews, Reviewers 1 and 3 did not suggest any further revisions, while Reviewer 2 offered two comments. We particularly appreciate the Reviewer 2 for his/her insights and comments, which were essential in improving our manuscript. We have carefully revised the manuscript according to the reviewers' comments and suggestions. The main changes are highlighted in **RED** in the **ANNOTATED VERSION**. Following is our point-by-point response to the reviewers' comments.

Best regards,
Yuzhong Peng
On behalf of all co-authors

To reviewer 2:

Comment 1: I am still concerned that the current revision does not fully rule out the possibility of over-optimistic model performance. In particular, I could not find the corresponding results or figure that would help evaluate the relationship between prediction accuracy and false negatives more clearly. Without such evidence, the reported performance still appears somewhat too good to be fully convincing. I therefore encourage the authors to provide the relevant analysis, figure, or discussion to better demonstrate that the model is not overfitted and that the predictive performance is robust. Providing additional validation (e.g., cross-validation results, independent test sets, or robustness analyses) would further strengthen confidence in the reported performance.

Response 1: Thank you for your insights and suggestion. To make the changes easier to identify where necessary, I have decomposed **Comment 1** into the sub-comments numbered as follows:

1.1 I could not find the corresponding results or figure that would help evaluate the relationship between prediction accuracy and false negatives more clearly. Without such evidence, the reported performance still appears somewhat too good to be fully convincing.

1.2 I therefore encourage the authors to provide the relevant analysis, figure, or discussion to better demonstrate that the model is not overfitted and that the predictive performance is robust. Providing additional validation (e.g., cross-validation results, independent test sets, or robustness analyses) would further strengthen confidence in the reported performance.

Response 1.1: Since the ROC curve is essentially a graphical representation of the relationship between the false positive rate and the accuracy rate, it illustrates the trade-off between accuracy and the false negative rate as the latter increases. To help you more clearly evaluate the relationship between prediction accuracy and the false negative rate, we have plotted the ROC curves of the proposed model on three datasets, as shown below.

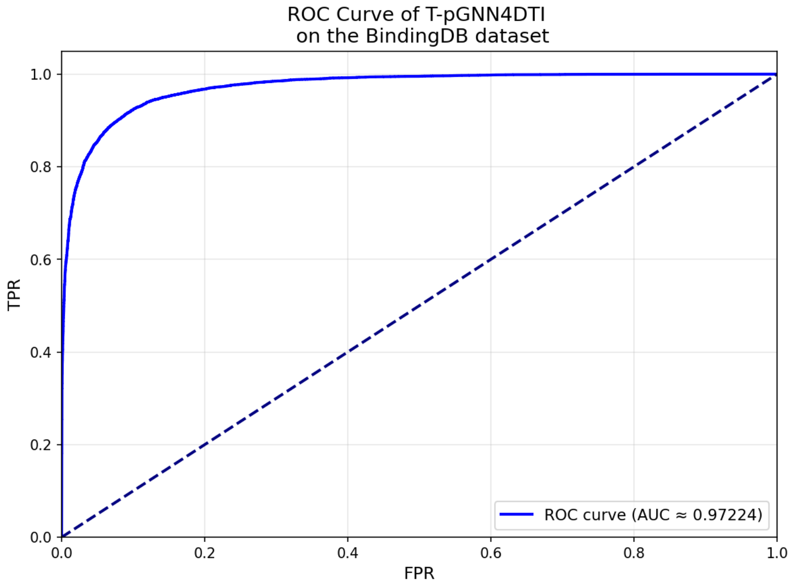
ROC Curve of T-pGNN4DTI
on the BindingDB dataset
TPR
FPR
0.0
0.2
0.4
0.6
0.8
1.0
ROC curve (AUC ≈ 0.97224)

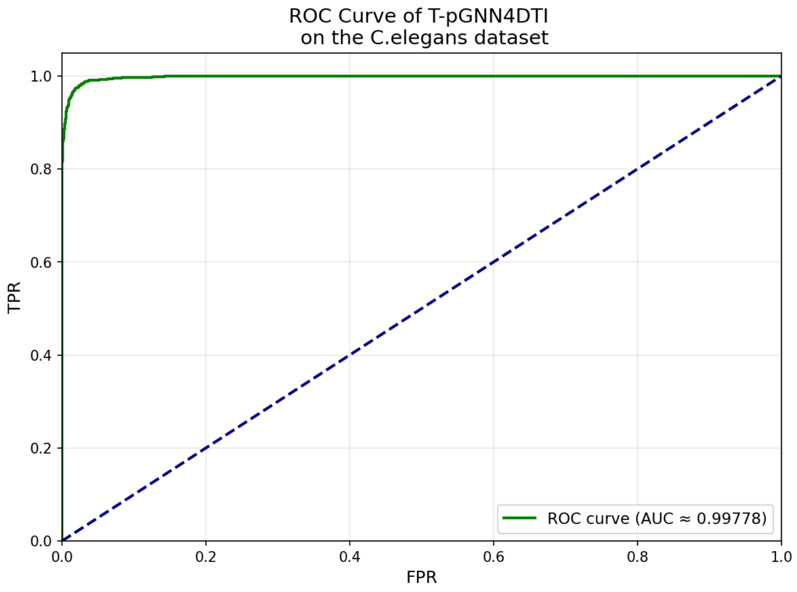
ROC Curve of T-pGNN4DTI
on the C.elegans dataset
TPR
FPR
0.0
0.2
0.4
0.6
0.8
1.0
ROC curve (AUC ≈ 0.99778)

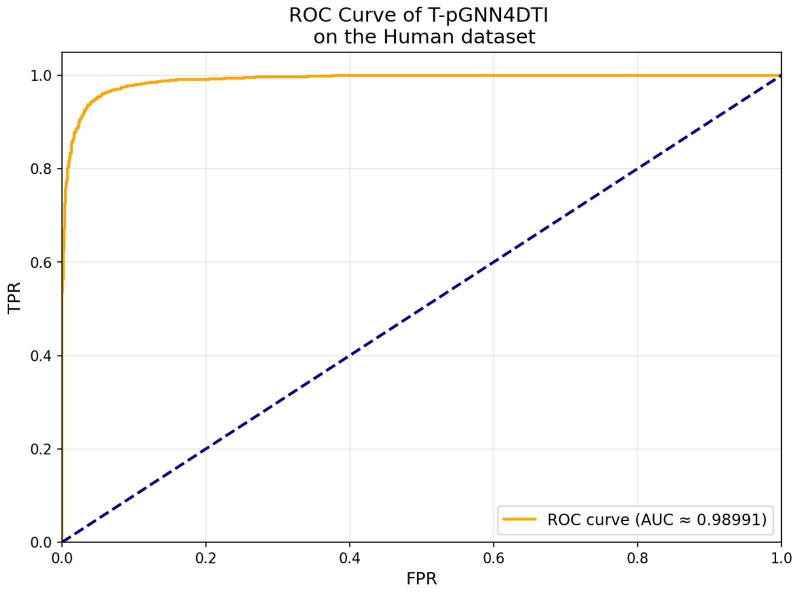
ROC Curve of T-pGNN4DTI
on the Human dataset
TPR
FPR
0.0
0.2
0.4
0.6
0.8
1.0
ROC curve (AUC ≈ 0.98991)

Response 1.2: Thank you for your comments and suggestion. Here, we provide additional evidence to demonstrate and corroborate the excellent performance of our T-pGNN4DTI model. We hope these supporting materials will further strengthen your confidence in the results reported in our paper.

Firstly, following your suggestion, we have conducted 5-Fold Cross-Validation experiments to evaluate T-pGNN4DTI against the baselines. All results are are obtained from our experiments using the same hyperparameter setting as reported in our paper, as shown in the following Table 1. Though all modelsexhibited some performance degradation under the 5-Fold Cross-Validation, our T-pGNN4DTI still outperforms the compared models.The results reveal that T-pGNN4DTI exhibits a certain degree of robustness.

Table 1 Perfomance comparison of 5-Fold Cross-Validation experiments

**Method**	**BindingDB**		***C.elegans***		***Huaman***	
	AUC	PRC	AUC	PRC	AUC	PRC
GCN	0.911±0.016	0.902±0.011	0.966±0.009	0.929±0.009	0.937±0.019	0.885±0.007
CF-DTI	0.959±0.006	0.951±0.005	0.988±0.005	0.989±0.004	0.981±0.006	0.980±0.003
$SAG_{hierarchical}$	0.958±0.005	0.958±0.008	0.990±0.005	0.990±0.004	0.979±0.005	0.977±0.007
FusionDTI	0.967±0.005	0.968±0.004	0.991±0.005	0.993±0.004	0.980±0.004	0.978±0.006
T-pGNN4DTI(our)	**0.969**±0.003	**0.970**±0.003	**0.994**±0.004	**0.995**±0.003	**0.985**±0.005	**0.985**±0.005

Secondly, we have plotted the loss curves showing the evolution of our model's training and validation losses on the BD dataset, as follows. The loss curves reveal that our model shows no significant evidence of overfitting.

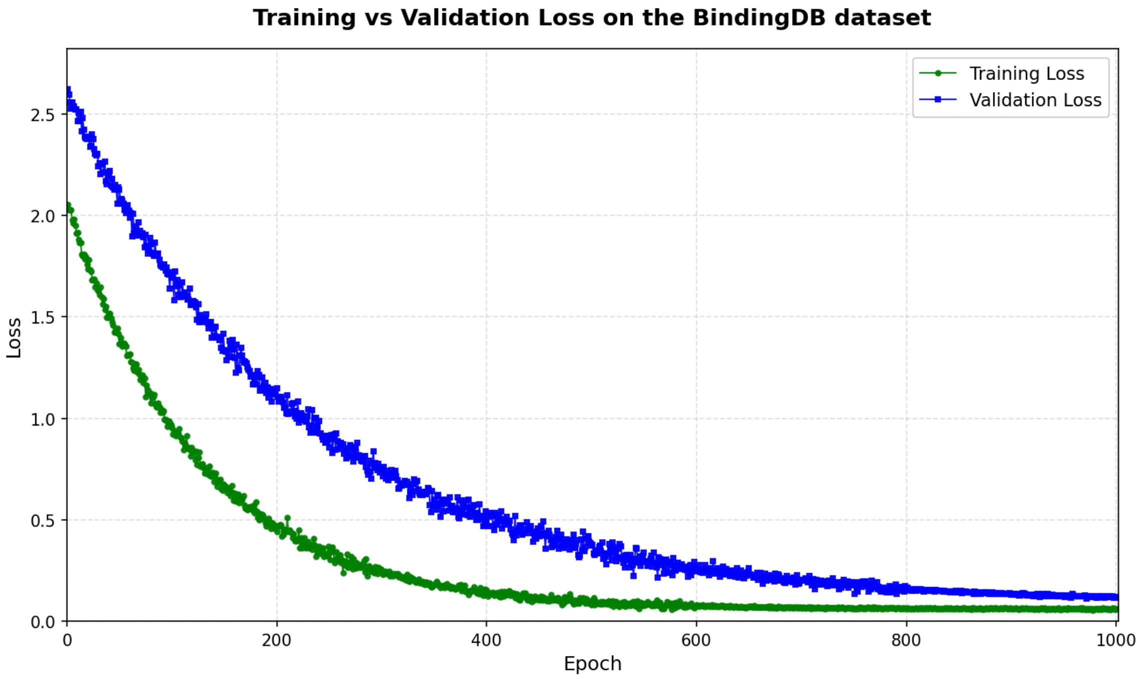

Thirdly, following your suggestion, we have conducted an experiment using a independent test set to evaluate the generalization ability of T-pGNN4DTI in real-world scenarios. The Independent test set consists of 2746 samples, sourced from the BioSNAP datasets, following the reference [1].

These drug-target pairs were completely excluded from the model training process, creating an "unseen-pair" scenario. All results are averaged over three independent runs for each model, as shown in the following Table 2. Note that the performance results for GCN, $SAG_{hierarchical}$, FusionDTI, and T-pGNN4DTI are obtained from our experiments, while those for GraphDTA, DrugBAN, AMMVF-DTI, and CF-DTI are cited from the reference [1]. Though all models experience a noticeable performance drop under the unseen-pair setting, our T-pGNN4DTI still outperforms the comparison models significantly.The results indicate that T-pGNN4DTI exhibits relatively good generalization performance.

Table 2 Perfomance comparison of the comparative models under the useen pair

**Method**	**AUC**	**PRC**
GraphDTA[1]	0.648	0.690
DrugBAN[1]	0.686	0.673
AMMVF-DTI[1]	0.772	0.764
CF-DTI[1]	0.784	0.800
GCN	0.581±0.007	0.598±0.006
$SAG_{hierarchical}$	0.741±0.003	0.737±0.003
FusionDTI	0.787±0.003	0.799±0.003
T-pGNN4DTI(our)	**0.793**±0.002	**0.808**±0.003

[1]Qian, Y., Wang, Q., Yin, L. et al. CF-DTI: coarse-to-fine feature extraction for enhanced drug-target interaction prediction. Health Information Science and Systems. 13, 55 (2025)

Comment 2: Second, at least one of the links provided in the manuscript appears not to be accessible. In particular, the GitHub link around line 538 could not be opened when I checked it. Please verify that all links are correct, active, and publicly accessible, as this is important for reproducibility and for allowing readers and reviewers to examine the associated code or resources.

Response 2: Thank you very much for your comment. We have carefully checked and tested the source code link in the GitHub and all other links to ensure that they are correct, active, and publicly accessible. We have investigated and determined that the GitHub link around line 538 could not be opened because our original GitHub account had been restricted. We have now created a new GitHub account and uploaded our source code and data to it. The link is: https://github.com/jedison2026/T-pGNN4DTI.This link has been updated in the revision (see the "Data and code availability" section). All other links are accessible as usual.
