# Supplementary material for: T-pGNN4DTI: Towards better drug-target interactions prediction using Global Self-attentive Pooled Graph Convolutional Networks and protein pre-training Models
Source: PLoS One. 2026 Jul 13;21(7):e0352250. doi: 10.1371/journal.pone.0352250 (PMC13362149; doi:10.1371/journal.pone.0352250)
Supplement: Attachment — Submitted filename: Response to Reviewers.docx [file pone.0352250.s005.docx]

Dear reviewers，

Thank you very much for your comments and professional advice. We sincerely appreciate the editors and reviewers for their invaluable insights and suggestions, which were essential in improving our manuscript. We have carefully revised the manuscript according to the reviewers' comments and suggestions. The main changes are highlighted in **RED** and **BLUE** in the **ANNOTATED VERSION**. Following is our point-by-point response to the reviewers' comments.

Best regards,

Yuzhong Peng

On behalf of all co-authors

**To reviewer 1:**

**Comment 1**: Unfortunately, the paper lacks the GitHub link for the dataset as well as references to the benchmarking datasets. Therefore, it is hard to evaluate their suitability for the approach. With regard to the benchmarking datasets, I would also expect a more detailed description of these datasets. What kind of data is in there and how were these datasets generated? Which limitations might be associated with their collection and how were positive and negative samples determined? Depending on the collection method, the benchmarking datasets might also include many false positive interactions. These limitations of the benchmarking datasets also needs to be discussed.

**Response 1:** Thank you for your valuable suggestions. To make the changes easier to identify where necessary, I have decomposed **Comment 1** into the sub-comments numbered as follows:

**1.1** The paper lacks the GitHub link for the dataset as well as references to the benchmarking datasets.

**1.2** I would also expect a more detailed description of these datasets, including the following issues: What kind of data is in there and how were these datasets generated? Which limitations might be associated with their collection and how were positive and negative samples determined?

**1.3** Depending on the collection method, the benchmarking datasets might also include many false positive interactions. These limitations of the benchmarking datasets also need to be discussed.

According to your suggestions, we have tried our best to revise our manuscript to meet your suggestions, as follows:

***Response 1.1:*** We have added a Section “Data and code availability” to give source for data and code availability.

***Response 1.2:*** We have added a more detailed description of the benchmarking datasets in Section “Benchmark datasets” to meet your suggestions. The added detailed description mainly includes: “The dataset employed in this study follows TransformerCPI~\cite{chen2020transformercpi}, drawing from three publicly available benchmark datasets widely recognized in the DTA prediction field in recent years\cite{Zhou2015Improving, Zhang2023A}, including the BindingDB, *Human*, and *C.elegans* datasets. Positive samples for the *Human* and *C.elegans* datasets were derived from positive drug-protein interaction (CPI) pairs in DrugBank 4.1 and Matador~\cite{chen2020transformercpi}, with high-confidence negative samples generated through the negative CPI screening framework ~\cite{Zhou2015Improving}. A more detailed introduction to the three benchmark datasets is given below.

BindingDB is a publicly accessible large-scale database measuring binding affinities, primarily focusing on interactions between proteins considered drug targets and drug-like small molecules~\cite{BindingDB}. The BindingDB dataset used in this work was obtained from TransformerCPI~\cite{chen2020transformercpi}, containing 39747 positive interactions (involving 1696 protein targets and 53253 small molecules) and 31218 negative samples. The *C.elegans* dataset contains extensive information on gene, protein, and compound interactions in the nematode Caenorhabditis elegans, including 4000 positive interactions between 1434 unique compounds and 2,504 unique proteins specific to the *C.elegans* species. The *Human* dataset contains 852 drugs and 1052 proteins, covering 3369 interaction pairs.”

***Response 1.3:*** We have added a discussion of the benchmarking datasets’ limitations in the “Discussion” section to address your suggestions. The added detailed description mainly includes:

“However, the benchmarking datasets are not ideal due to limitations in data collection methods and experimental conditions. For example, most drugs in the *Human* and BindingDB dataset only occur in one class, potentially introducing bias into feature learning. The relatively small sample size of compound-protein interactions (CPIs) in *Human* and *C.elegans* may limit the training of complex deep learning models. Furthermore, negative samples are generated by algorithms that may introduce undetectable noise~\cite{Zhou2015Improving}, necessitating biological experimental corrections by the scientific community.”

**Comment 2**:	The authors compared their method on the Human dataset against ten methods while the C. elegans and BindingDB dataset was only compared against eight and six methods. What is the reason for the missing comparisons? In this sense, I would expect to include the missing comparisons or at least discuss why they are missing.

**Response 2:** Thank you for your comment. The reason for the missing comparisons is as follows: the comparative data in the manuscript were partially reproduced by the authors (including GCN, FusionDTI, and two variant models of SAG) and partially cited from relevant literatures. However, some original papers did not cover all benchmark datasets or had differences in experimental settings and evaluation metrics, making fair comparisons impossible and thus not included in our manuscript. For instance, the original papers for DrugVQA and AMMVF-DTI present results for the *Human* dataset but omit results for the *C. elegans* and BindingDB datasets. Consequently, these two methods are not compared in the *C. elegans* and BindingDB dataset experiments, resulting in a difference in the number of methods compared across datasets. These resulted in an inconsistent number of comparison methods across different datasets.

**Comment 3**: The authors compare themselves to methods that are published up to 2023, but state that their method is the new state of the art. Were there no further methods published between 2023 and 2025?

**Response 3:** Thank you for your valuable comment. We have added 5 state-of-the-art methods in the revised manuscript, including two (IMAEN and MGNDTI) published in 2024 , two (CF-DTI and FusionDTI) published in 2025, and one (TopoPharmDTI) published in 2026. As demonstrated in Table 3~5. All the comparative SOTA models underperformed T-pGNN4DTI. More detailed can be seen in Sections “Baseline models” and “Comparisons results” in revised manuscript. This demonstrates that T-pGNN4DTI is an effective method for improving DTI prediction.

**Comment 4**: A critical evaluation of the approach itself and the applied benchmark dataset would be beneficial for the discussion. Are the mis-identified drug-target interactions similar between the different methods and do these sequences or drugs have something in common?

**Response 4:** Thank you for your insightful comment. Following your suggestion, we systematically analyzed the misclassified drug-target interactions in the GCN and FusionDTI models reproduced in this paper and our proposed model. We found that (1) significant overlap exists among the misclassified drug-target interaction samples across the three models. Many samples misclassified by GCN and FusionDTI were also misclassified by our proposed model. This may be because some key biological features of these samples in DTIs can not be fully exploited by these deep learning models; (2) most misclassified samples within each model did not exhibit strong similarities in sequence or drug structure. Moving forward, we will employ more advanced methods and techniques to investigate whether more subtle common features exist, and we will incorporate your suggestions into our future research endeavors.

**Comment 5**: A reduced tone would be beneficial for the abstract. Of course machine learning can support the discovery of drug-interactions but they might only reduce laboratory efforts to check only the most promising interactions.

**Response 5:** Thank you very much for your insightful suggestions. We have revised the phrasing of the third and final sentences in the abstract according to your suggestions. i.g., “Machine learning-based methods can rapidly predict DTI using only computer algorithmic models, allowing researchers to validate only the most promising interactions through biochemical experiments. This holds promise for effectively addressing the current challenges of lengthy development cycles and high costs in new drug development.”,“It provides a new way of thinking to help solve the DTI-related problems.”

**Comment 6**: The authors report that their method learns more meaningful features of drug molecules by paying more attention to information features of certain important atomic nodes of the molecular structure. This raises the question for me whether there are common patterns in these molecules that are responsible for this effect. Similarly, the assignment of weights to nodes (line 315), including recommendations on how to assign these weights, requires a more detailed description, including the consequences.

**Response 6**: Thank you for your valuable suggestions. To make the changes easier to identify where necessary, I have decomposed **Comment 6** into the sub-comments numbered as follows:

**6.1** This raises the question for me whether there are common patterns in these molecules that are responsible for this effect.

**6.2** Similarly, the assignment of weights to nodes (line 315), including recommendations on how to assign these weights, requires a more detailed description, including the consequences.

According to your suggestions, we have tried our best to revise our manuscript to meet your suggestions, as follows:

***Response 6.1:*** Yes, common patterns among molecules in the training set are captured by the attention mechanism algorithm, thereby inducing this effect.

***Response 6.2:*** We have added detailed explanations on how these weights are assigned in the revision, such as lines 264-278 of the “Drug Feature Extraction” section. Specific examples are provided in the “T-pGNN4DTI Case Studies and Interpretability” section, where you can further understand the weight assignment method and its effects (as shown in Figure 6).

**Comment 7**: All figure legends require a more detailed description. A title alone is not enough to enable the reader to understand what is depicted in the figure.

**Response 7:** Thank you very much for your valuable suggestions. We have supplemented each figure legend with a more detailed description.

**Comment 8**: Figure 4 is blurred and needs to be replaced.

**Response 8:** Thank you very much for your valuable suggestions. We have redrawn a clearer version of Figure 4.

**Comment 9**: Avoid colloquial formulations, e.g., and so on (line 119).

**Response 9:** Thank you very much for your valuable suggestions. We have re-optimized the language expression of the manuscript to make it more standardized, smoother, and logically more rigorous in the revision. e.g., lines 90-92, 139-140,142-144, 136-137, 147-149, 175-176, 218-223, 235-236,238-243, 247-252, 263-265,293,303,479-480,502-503 and so on.

**Comment 10**: According to standard naming conventions, the names of organisms must be in italics (e.g., C. elegans).

**Response 10:** Thank you very much for your insightful suggestions. We have adopted your suggestion and italicized all organism names (including *C. elegans* and *humans*) in accordance with standard naming conventions.

**To reviewer 2:**

**Comment 1**: Insufficient explanation of the methodological novelty and integration

The manuscript introduces a global self-attention pooling module and several combined components (graph neural network, protein pre-training model, and feature fusion), yet the originality and integration of these elements remain unclear. Please clarify which parts of the framework are newly developed and which are adapted from prior studies. The authors should also explain why the global self-attention pooling mechanism was introduced, how it differs from existing pooling strategies, and how it affects downstream performance. A concise schematic or ablation focusing on this component’s contribution would improve clarity.

**Response 1:** Thanks for your valuable suggestions. The reviewers commented on the issue “Insufficient explanation of the methodological novelty and integration”. To make the changes easier to identify where necessary, I have decomposed **Comment 1** into the sub-comments numbered as follows:

**1.1** The originality and integration of these elements remain unclear. Please clarify which parts of the framework are newly developed and which are adapted from prior studies.

**1.2** The authors should also explain why the global self-attention pooling mechanism was introduced and how it differs from existing pooling strategies.

**1.3** How it affects downstream performance.

**1.4** A concise schematic or ablation focusing on this component’s contribution would improve clarity.

***Response 1.1:*** Thank you for pointing this out. Our T-pGNN4DTI is not a simple stacking of existing components. Instead, our T-pGNN4DTI proposes an integrative innovation strategy to tackle the challenge of drug-target interaction prediction. This strategy involves the synergistic and complementary integration of three major components, including protein feature learning, compound feature learning, and feature fusion with pattern recognition.

It integrates three complementary and synergistic components (including a protein pre-training model, global attention pooling graph convolutional neural networks, and concatenation + MLP) and end-to-end training. This high-performance drug-target interaction prediction method embodies an innovative tripartite integration mechanism of “generalization-focus-efficiency”. Compared to existing single-technique approaches (e.g., relying solely on pre-trained models, recurrent neural networks, or graph neural networks) or fusion techniques (e.g., LSTM+CNN, LSTM+GAT, protein pre-trained models + compound pre-trained models, or other complex fusion architectures), our methodology's core innovation lies in the complementary synergy of its components and end-to-end optimization. Together, these overcome bottlenecks of data sparsity, feature fragmentation, and computational efficiency, achieving DTI prediction performance that surpasses existing methods. See the SOTA comparison results (Tables 3–5) and ablation study results (Table 6) in the “Experimental Results” section of the revision.

Specifically, existing protein sequence learning models (e.g., TransformerCPI) heavily rely on labeled sample data. Our T-pGNN4DTI leverages pre-trained protein models to learn evolutionary conservation, sequence patterns, and structural priors, generating robust, generalized protein features that significantly reduce dependence on labeled data and address data sparsity. Existing standard graph neural networks and GATs (e.g., GraphDTA) employ global average pooling, overlooking local interaction details between compounds and proteins. Our T-pGNN4DTI uses globally attentive pooling graph networks to learn compound features. By dynamically weighting node importance (e.g., atoms, functional groups, and substructures), it enhances focus on and capture of critical compound substructures during drug-target interactions, improving interpretability. It demonstrates significant advantages over traditional graph neural networks in noise robustness, structural fault tolerance, and data missing compensation. Existing feature fusion methods often involve computationally complex operations (e.g., DMFF-DTA), resulting in high computational overhead that hinders scalability. Our T-pGNN4DTI employs feature concatenation and MLP to integrate and align learned protein and compound features, ensuring lossless transmission of sequence and structural information. This effectively combines local features of drugs and targets, successfully mining key biological characteristics in drug-target interactions and enabling lightweight, efficient drug-target interaction prediction.

In summary, although both the PTR and self-attention pooling neural networks in our methodology draw inspiration from existing methods, the core components of our framework synergistically complement each other to form a closed-loop system, collectively achieving a synergistic effect where the whole is greater than the sum of its parts (“1+1+1>3”).

***Response 1.2:*** Thank you for pointing this out. Graph pooling methods can be categorized into three types: topology-based pooling, global pooling, and hierarchical pooling. Topology-based graph pooling methods typically only consider the graph's topological structure, while global graph pooling methods only consider the graph's features. Among hierarchical graph pooling methods, the classic DIFFPOOL approach learns hierarchical representations of the graph, enabling graph neural networks to converge end-to-end and produce scaled-down graphs. However, it suffers from quadratic space complexity. The gPool method addresses this complexity issue but neglects the graph's topological structure.

The SAGPool method employed in this work is the first to utilize self-attention for graph pooling while achieving high performance. It combines the advantages of previous approaches: hierarchical pooling, simultaneous consideration of node features and graph topology, reasonable computational complexity, and end-to-end representation learning. Benefiting from its dynamic weight allocation and hierarchical feature extraction techniques, it demonstrates significant advantages over traditional graph neural networks in noise robustness, structural fault tolerance, and data missing compensation. This is highly significant for molecular graph feature learning scenarios, as tasks involving molecular graph learning critically depend on the graph's topological information, multi-scale hierarchical structure, and node feature information.

***Response 1.3:*** Thank you for pointing this out. Attention-based graph pooling introduces an attention mechanism that enables graph neural networks to dynamically assign importance weights to different nodes during pooling. This allows the model to focus on the most informative subgraphs, significantly enhancing its representational capacity for graph data and task performance. It is a key technology for achieving efficient, adaptive hierarchical learning. This has been validated in some published studies, such as “Lee J, Lee I, Kang J. Self-attention graph pooling[C]//International conference on machine learning. pmlr, 2019: 3734-3743.” and “Xu Y, Wang J, Guang M, et al. Graph multi-convolution and attention pooling for graph classification[J]. IEEE Transactions on Pattern Analysis and Machine Intelligence, 2024, 46(12): 10546-10557.“ and ”Tang J, Miao Y, Xia Y, et al. A multi-scale pooling attention-based graph attention network for remaining useful life prediction[J]. IEEE Transactions on Instrumentation and Measurement, 2025."

***Response 1.4:*** Thank you for pointing this out. We have added ablation comparison experiments, replacing our PTR with two protein language models (ESM-1v and ProteinBERT), and attempting to replace our GSAP-GCN with BiLSTM. When presented alongside the original ablation results, these clearly demonstrate the synergistic performance of our components and their contributions within T-pGNN4DTI. We have made every effort to enhance the clarity of the entire text and address errors and inconsistencies. We hope this revised version meets the requirements.

**Comment 2**: Missing dataset and unavailable code

The manuscript does not provide access to the datasets used in the study, nor does it include a GitHub or other code repository link. Data and code availability are essential for ensuring reproducibility and independent verification.

**Response 2:** Thank you for your comment. We have added a section on “Data and code availability” to give sources for data and code availability. The demo code of T-pGNN4DTI was released on https://github.com/jedison2026/T-pGNN4DTI.

**Comment 3**: Lack of statistical rigor

The current results section lacks sufficient statistical validation. The manuscript reports single-run values for AUC, Precision, and Recall, but does not show whether these results are stable or statistically significant.

• No information is given on how many times each experiment was repeated.

• No averages, standard deviations, or confidence intervals are reported to indicate result variability.

• No statistical tests are conducted to verify that improvements over baselines are meaningful.

The authors should, report mean ± SD or confidence intervals, specify the number of samples used, and perform significance testing when comparing against other methods.

**Response 3:** Thank you for your insightful comments. First, we apologize for not describing in the experimental setup that our paper's results represent the average of 10 repeat runs with different random seeds, along with certain statistical details raised by reviewers. This omission prevented reviewers from accurately understanding our methodology and experimental results. We have now supplemented the revised manuscript with our experimental statistics, including key information such as 10 runs with different random seeds, reporting mean ± SD, and results of statistical tests. More detailed in the last paragraph of the “Baseline Models” section and the SOTA comparison results (Tables 3-5) in the “Comparison Results” section.

**Comment 4**: Hyperparameter configuration and potential overfitting risk

In most comparable studies, the number of training epochs does not exceed 500, and dropout rates typically range from 0.2 to 0.5 (e.g., GraphDTA, SAG-DTA, CPGL, TransformerCPI). In contrast, the present study trains for 1000 epochs with a very low dropout rate of 0.1, yet reports nearly perfect performance (AUC ≈ 0.998). This combination suggests a substantial risk of overfitting

To ensure the reported performance is reliable and not a result of overfitting, the authors should provide learning curves showing training and validation loss across epochs, or at least describe the use of early-stopping criteria. Additionally, for fair comparison, baseline methods should be trained under similar or equivalent hyperparameter settings.

**Response 4:** Thanks for your valuable suggestions. The reviewers commented on the issue “Hyperparameter configuration and potential overfitting risk”. To make the changes easier to identify where necessary, I have decomposed **Comment 4** into the sub-comments numbered as follows:

**4.1** The present study trains for 1000 epochs with a very low dropout rate of 0.1, yet reports nearly perfect performance (AUC ≈ 0.998). This combination suggests a substantial risk of overfitting.

**4.2** To ensure the reported performance is reliable and not a result of overfitting, the authors should provide learning curves showing training and validation loss across epochs, or at least describe the use of early-stopping criteria.

**4.3** Additionally, for fair comparison, baseline methods should be trained under similar or equivalent hyperparameter settings.

***Response 4.1:*** Thank you for your insightful comments. We understand that excessive training epochs can lead to model overfitting. We also note that GraphDTA, SAG-DTA, CPGL, and TransformerCPI typically employ around 500 training epochs with dropout rates ranging from 0.2 to 0.5. However, our experiments revealed that our T-pGNN4DTI continues to improve validation set prediction performance beyond 500 epochs on corresponding benchmark datasets and partitioning methods in this work.

In our experiment, we observed differences in the optimal number of training epochs across datasets: BindingDB, Human, and C. elegans required 1000, 528, and 689, respectively. For consistency, we set the highest value of 1000 epochs as the default parameter. Early-stopping criteria were employed to determine the actual optimal number of training epochs for each dataset, preventing model overfitting caused by excessively high training epochs.

***Response 4.2:*** Thank you for your insightful comments. We apologize for the omission of details about our use of early-stopping criteria in the experimental setup, which may have confused reviewers in accurately understanding our experimental procedures. We have now supplemented Table 2 with relevant information regarding the early-stopping criteria employed in our experiments. The parameter settings for the early-stopping criteria employed in our experiments are as follows: monitor="AUC", patience=25, min_delta=0.0001, restore_best_weights=True.

***Response 4.3:*** Thank you for your comments. For fair comparison, we employed the same dataset partitioning method and evaluation metrics. However, we did not use similar or equivalent hyperparameter settings for our model and baseline methods. This is because we believe the authors of the baseline models have already optimized their parameters to the best of their knowledge in their published works. The parameters described in their papers represent the optimal parameter combinations for their models. Altering the parameter settings of the baseline methods or our own model could potentially lead to suboptimal performance.

**Comment 5**: Language and formatting

In lines 283–294 and 299, the improvements are expressed in decimals (e.g., “0.015 higher”), whereas in lines 274–282 and 295–298 they are expressed in percentages (e.g., “0.52% to 78.82% higher”).

**Response 5:** Thank you for your insightful comments and constructive suggestions, which are invaluable for improving our paper. We have revised the text to adopt a consistent language style, i.e., expressed in percentages. (e.g., “0.52% to 78.82% higher”), as shown in the “Comparison Results” section.

**To reviewer 3:**

**Comment 1**: The T-pGNN4DTI model proposed in the paper is essentially a combination of existing global self-attention pooling technology and pre-trained protein language models, but the paper fails to adequately explain why this combination is particularly suitable for DTI prediction. It is suggested that the authors elaborate on why global self-attention pooling can effectively capture atoms critical to interactions in drug molecules. and why pre-trained protein language models better represent protein binding site features, from the perspective of biochemical mechanisms of drug-target interactions. The authors could introduce specific drug-target interaction cases to analyze how the model identifies key interaction sites, thereby providing a stronger theoretical foundation for the method.

**Response 1:** Thanks for your valuable suggestions. The reviewers commented on the issue “the paper fails to adequately explain why this combination is particularly suitable for DTI prediction”. To make the changes easier to identify where necessary, I have decomposed **Comment 1** into the sub-comments numbered as follows:

1.1 It is suggested that the authors elaborate on why global self-attention pooling can effectively capture atoms critical to interactions in drug molecules.

1.2 Why pre-trained protein language models better represent protein binding site features, from the perspective of biochemical mechanisms of drug-target interactions.

1.3 The authors could introduce specific drug-target interaction cases to analyze how the model identifies key interaction sites, thereby providing a stronger theoretical foundation for the method.

***Response 1.1:*** Thank you for your comments. SAGPool is a method that employs self-attention for hierarchical graph pooling to achieve high performance. It simultaneously processes the topological structure information, multi-scale hierarchical information, and node feature information of learned graphs, as demonstrated by several published studies, such as “Lee J, Lee I, Kang J. Self-attention graph pooling[C]//International conference on machine learning. pmlr, 2019: 3734-3743.” and “Xu Y, Wang J, Guang M, et al. Graph multi-convolution and attention pooling for graph classification[J]. IEEE Transactions on Pattern Analysis and Machine Intelligence, 2024, 46(12): 10546-10557.“ and ”Tang J, Miao Y, Xia Y, et al. A multi-scale pooling attention-based graph attention network for remaining useful life prediction[J]. IEEE Transactions on Instrumentation and Measurement, 2025."

In molecular graph learning tasks, the topological structure, multi-scale hierarchical information, and node features of molecular graphs are all crucial feature elements. Therefore, the characteristics and advantages demonstrated by SAGPool are highly relevant to scenarios involving molecular graph feature learning, such as DTI prediction. These include simultaneously considering node features and graph topology, enabling hierarchical pooling of molecular graphs for multi-level information aggregation, achieving reasonable computational complexity, and facilitating end-to-end representation learning. This enables the SAGPool method to effectively capture atoms critical to interactions in drug molecules.

***Response 1.2:*** Thank you for your comments. Because the pre-trained protein model in T-pGNN4DTI can learn protein evolutionary conservation, sequence patterns, and structural priors, generating highly generalizable universal protein features. This not only significantly reduces reliance on labeled sample data and addresses data sparsity issues, but also synergistically complements the global attention pooling graph network and MLP. Consequently, it effectively integrates and aligns learned protein features with compound features, ensuring lossless transmission of sequence and structural information. Effectively integrating local features of drugs and targets allows for the thorough exploration of key biological characteristics within drug-target interactions.

***Response 1.3:*** Thank you for your valuable comments. We have added a section titled “Case and Interpretability for T-pGNN4DTI” in the revision using Ritonavir as an example to analyze and demonstrate how T-pGNN4DTI identifies key interaction points from the GSAP-GCN perspective.

**Comment 2**:The description of the PTR pre-training model in the paper lacks sufficient detail and fails to explain why PTR was chosen over other widely used protein pre-training models.The authors are advised to supplement the complete technical details of the PTR model, including the scale and source of the pre-training corpus, model architecture parameters, and training strategies. Additionally, comparative experiments with different pre-trained protein models should be conducted to analyze performance differences and their causes in DTI prediction tasks.Visualization analyses could also be employed to demonstrate how PTR captures key regions in protein sequences related to drug binding, thereby validating its applicability in DTI tasks.

**Response 2:** Thank you for your valuable suggestions. The reviewers commented on the issue, “the paper lacks sufficient detail and fails to explain why PTR was chosen over other widely used protein pre-training models”. To make the changes easier to identify where necessary, I have decomposed **Comment 2** into the sub-comments numbered as follows:

2.1 The authors are advised to supplement the complete technical details of the PTR model, including the scale and source of the pre-training corpus, model architecture parameters, and training strategies.

2.2 Comparative experiments with different pre-trained protein models should be conducted to analyze performance differences and their causes in DTI prediction tasks.

2.3 Visualization analyses could also be employed to demonstrate how PTR captures key regions in protein sequences related to drug binding, thereby validating its applicability in DTI tasks.

***Response 2.1:*** Thank you for your valuable comments. We have added descriptions of some key PTR technologies in paragraphs 2~3 of the “Feature of the protein extraction module” section, mainly including: “The model is a bidirectional Masked language model, enabling it to observe information (context) near a specific residue or motif and thereby predict that residue or motif. It comprises an encoder module and a decoder module, which are both based on multi-head attention mechanisms and feedforward neural networks, as illustrated in Fig.3. The work process of PTR can be divided into two stages: the pre-training stage and the application stage. The former stage provides the latter stage with a large number of model parameters and contextual relationships within sequences”,

“Finally, the embedding vector of the protein sequence $IV=\{IV_1,IV_2,\cdots,OV_L \}$ is used as input to the PTR model. In the PTR model, $IV$ is first fed into the multi-head self-attention layer of the encoder. Within each head of the multi-head self-attention layer, we project the protein features onto query, key, and value vector spaces via projection matrices. The output of each attention head is the result of scaled dot-product attention, as follows,

\begin{equation}

\begin{aligned}

Attention(Q,K,V ) &= \boldsymbol{a} V \\

&=softmax(QK^T/\sqrt d_k) V

\end{aligned}

\label{eq:30}

\end{equation}

where, $\boldsymbol{a}$ is the self-attention score matrix, which is an attention weight matrix that follows a probability distribution; $d_k$ is the scaling factor (typically set to the dimension of the key vector); $Q$, $K$, and $V$ represent the query matrix, key matrix, and value matrix, respectively. They are obtained by multiplying the input features by their corresponding weight matrices. The encoder runs the scaled dot-product attention mechanism demonstrated by Eq.(\ref{eq:30}) $M$ (i.e., the number of heads) times with $M$ different queries in parallel and concatenates their outputs. In this way, the self-attention mechanism explicitly constructs pairwise interactions ($QK^T$) between all positions in the sequence, enabling the model to represent interactions between residues directly.”,

“ Additionally, the PTR model adopted a pre-training strategy similar to AlphaFold3.”,

“The pre-training corpus is downloaded from RCSB PDB (https://www.rcsb.org, including more than 250000 structures from the PDB archive and 1060000 Computed Structure Models), as well as the protein Swiss-Prot database (https://www.uniprot.org, including about 570000 structures).”

The more details of the training strategies of the PTR model (Supplementary information S1.1) are described as follows:

The PTR model adopted a pre-training strategy similar to AlphaFold3. Training comprises four stages: First, the model is trained with random initialization using a sequence cropping size of 384. Subsequently, fine-tuning is performed with a cropping size of 640 based on the weights from the first stage, followed by secondary fine-tuning with a cropping size of 768. During the first fine-tuning stage, the smooth LLDT loss is disabled, and weights from the disordered protein PDB distillation set are reduced. However, unmasked diffusion loss for non-protein chains within this distillation set is now enabled. In the second fine-tuning stage, the transcription factor distillation set is additionally enabled, and weights from the disordered protein PDB distillation set are restored to their original levels. Protein residues from the DNA motif distillation set were considered unresolved if their experimentally resolved probability prediction was below 0.9. In the third (final) fine-tuning stage, we removed all structure-based loss terms (diffusion and distribution map) during training and increased the maximum chain length to 50. All other settings remained consistent with the previous fine-tuning stage.

***Response 2.2:*** Thank you for your valuable comments. We have added the ablation comparison experiments replacing our PTR with ESM-1v and ProteinBERT, which confirmed the relative superiority of our PTR model, as shown in the “Ablation experiments and results” section of the revision.

***Response 2.3:*** Thank you for your valuable comments. We have added a new section titled “Case Studies and Interpretability of T-pGNN4DTI,” which analyzes the example of ritonavir to demonstrate how this model identifies key interaction points. This case reveals how ritonavir acts like a “key” that inserts into the protease active site. This not only helps elucidate the interaction mechanism between ritonavir and HIV-1 protease but also demonstrates that T-pGNN4DTI can automatically identify chemically meaningful interactions without manual feature engineering!

**Comment 3**: Although the authors emphasize that the global self-attention mechanism can identify key atomic nodes in molecular structures, the paper lacks detailed interpretability analysis to demonstrate this. There is no visualization of attention weight distributions, nor analysis of whether the atomic nodes focused on by the model correspond to actual drug-target binding sites. It is recommended that the authors add an interpretability analysis section to enhance the model's explainability and provide valuable insights for drug design.

**Response 3:** Thank you for your valuable suggestions. We have added a section titled “Case and Interpretability for T-pGNN4DTI,” using Ritonavir as an example to analyze and demonstrate how T-pGNN4DTI identifies key interaction points from the GSAP-GCN perspective. In this section, we detail and demonstrate how the attention mechanism of GSAP-GCN participates in learning molecular features from atomic-edge to motif, and from functional groups to the entire molecule. We also provide their attention scores (attention weight distributions). This explains how our model identifies key atomic nodes and motifs in molecular structures for drug-target interactions.

**Comment 4**: The paper does not provide computational complexity analysis or comparisons of training and inference times for the model. In practical applications, model efficiency is as important as accuracy, especially in large-scale virtual screening scenarios. Furthermore, the lack of discussion regarding the model's parameter count makes it difficult to assess its resource requirements compared to existing methods. It is recommended that the authors supplement the paper with a comprehensive analysis of computational efficiency.

**Response 4:** Thank you for your valuable suggestions. We have added a section titled “Time Complexity and Computational Efficiency” in the paper's supplementary materials to provide the computational complexity analysis. The main content is as follows:

**“S1.2 Time Complexity and Computational Efficiency**

To estimate the time complexity of our framework, we can first decompose the model and independently assess the time complexity of its three core components as follows:

**(1) Time Complexity of the** **PTR Model:**

Our PTR model is based on the Transformer architecture, where the following factors primarily determine its pre-training time complexity:

Sequence Length (𝑇): The number of amino acids in the protein sequence.

Hidden layer dimension (𝐷): Size of the feature vector.

Number of layers (𝐿): Number of layers in the Transformer encoder.

The core time complexity formula is 𝑂(𝐿⋅𝑇²⋅𝐷)

This complexity stems from the self-attention mechanism requiring computation of all amino acid pair relationships within the sequence (the 𝑇² term).

**(2) Time Complexity of GSAP-GCN:**

GSAP-GCN's time complexity is primarily determined by: the number of network layers *L_g_*, the number of edges 𝐸 in the molecular graph, the number of nodes (atoms) 𝑉 in the molecular graph, and the feature dimension *F* of the node features.

Therefore, the core time complexity formula for GSAP-GCN is *O*(*L_g_⋅*(*E⋅F²*) *+ N⋅F*)

**(3) Time Complexity of FNN:**

The time complexity of full connected FNN is primarily determined by the following factors: the dimension of protein features *D*, the dimension of compound features *F*, the number of hidden layers *L_f_* in the FFN, and the size of the output features *N_f_*.

The core time complexity formula is: *O(N_f_⋅*(*D+F*)*+*( *L_f_−1*)*⋅O*(*N_f_*))

To facilitate the construction of deep feedforward neural networks and complexity analysis, the input dimension of the intermediate hidden layer is typically set equal to its output dimension. That is, *N*= *D+F*. Therefore, the core time complexity can be simplified as: *O*(*L_f_⋅*(*D+F*))

**(4) Total Time Complexity and Computational Efficiency of T-pGNN4DTI:**

Since protein feature extraction and compound feature extraction are performed in parallel, the total time complexity is the sum of both plus the FFN component, as follows:

*O*(T-pGNN4DTI) = *O*(PTR) + *O*(GSAP-GCN) + *O*(FFN)

= 𝑂(𝐿⋅𝑇²⋅𝐷)+ *O*(*L_g_⋅*(*E⋅F²*) *+ N⋅F*)+ *O*(*L_f_⋅*(*D+F*))

Since *T* is much larger than *N*, the time complexity of **T-pGNN4DTI** primarily depends on the time complexity of the PTR model. In practical DTI prediction applications, *T* averages around 1000, *N* averages around 50, and the PTR model accounts for over 99% of the computational overhead. In our experiments, the GPU used was an NVIDIA RTX 4080 Ti, achieving an inference time of approximately 100 milliseconds per drug-target pair sample. Consequently, our**T-pGNN4DTI** can scale for DTI prediction in higher-performance GPU server cluster environments.**”**

**Comment 5**: It is recommended that the authors introduce more advanced methods for drug association prediction in the introduction, not limited to DTI research, focusing on summarizing innovative methods such as "Multi-view contrastive learning for drug-drug interaction event prediction" and "LLM-DDI: Leveraging Large Language Models for Drug-Drug Interaction Prediction on Biomedical Knowledge Graph." This would highlight the cutting-edge nature and scientific value of the authors' proposed method.

**Response 5:** Thank you for your suggestions. We have incorporated the two references you suggested into the introduction, namely references [9] and [28] in the revised manuscript, as indicated on lines 8 and 21 of the introduction.
