# Supplementary material for: T-pGNN4DTI: Towards better drug-target interactions prediction using Global Self-attentive Pooled Graph Convolutional Networks and protein pre-training Models
Source: PLoS One. 2026 Jul 13;21(7):e0352250. doi: 10.1371/journal.pone.0352250 (PMC13362149; doi:10.1371/journal.pone.0352250)
Supplement: Peer review history

# Peer review: Decision Letter 0

**DOI:** 10.1371/journal.pone.0352250.r001

**Authors:** Lun Hu

13 Jan 2026

PONE-D-25-56836

T-pGNN4DTI: Towards Better Drug-target Interactions Prediction Using Global Self-attentive Pooled Graph Convolutional Networks and Protein Pre-training Models

PLOS One

Dear Dr. Peng,

Thank you for submitting your manuscript to PLOS ONE. After careful consideration, we feel that it has merit but does not fully meet PLOS ONE’s publication criteria as it currently stands. Therefore, we invite you to submit a revised version of the manuscript that addresses the points raised during the review process.

Please submit your revised manuscript by Feb 27 2026 11:59PM. If you will need more time than this to complete your revisions, please reply to this message or contact the journal office at plosone@plos.org. When you're ready to submit your revision, log on to https://www.editorialmanager.com/pone/ and select the 'Submissions Needing Revision' folder to locate your manuscript file.

Please include the following items when submitting your revised manuscript:

- A letter that responds to each point raised by the academic editor and reviewer(s). You should upload this letter as a separate file labeled 'Response to Reviewers'.
- A marked-up copy of your manuscript that highlights changes made to the original version. You should upload this as a separate file labeled 'Revised Manuscript with Track Changes'.
- An unmarked version of your revised paper without tracked changes. You should upload this as a separate file labeled 'Manuscript'.

If you would like to make changes to your financial disclosure, please include your updated statement in your cover letter. Guidelines for resubmitting your figure files are available below the reviewer comments at the end of this letter.

If applicable, we recommend that you deposit your laboratory protocols in protocols.io to enhance the reproducibility of your results. Protocols.io assigns your protocol its own identifier (DOI) so that it can be cited independently in the future. For instructions see: https://journals.plos.org/plosone/s/submission-guidelines#loc-laboratory-protocols. Additionally, PLOS ONE offers an option for publishing peer-reviewed Lab Protocol articles, which describe protocols hosted on protocols.io. Read more information on sharing protocols at https://plos.org/protocols?utm_medium=editorial-email&utm_source=authorletters&utm_campaign=protocols.

We look forward to receiving your revised manuscript.

Kind regards,

Lun Hu

Academic Editor

PLOS One

Journal Requirements:

When submitting your revision, we need you to address these additional requirements.

1. Please ensure that your manuscript meets PLOS ONE's style requirements, including those for file naming. The PLOS ONE style templates can be found at

https://journals.plos.org/plosone/s/file?id=wjVg/PLOSOne_formatting_sample_main_body.pdf and

https://journals.plos.org/plosone/s/file?id=ba62/PLOSOne_formatting_sample_title_authors_affiliations.pdf.

2. Please note that PLOS One has specific guidelines on code sharing for submissions in which author-generated code underpins the findings in the manuscript. In these cases, we expect all author-generated code to be made available without restrictions upon publication of the work. Please review our guidelines at https://journals.plos.org/plosone/s/materials-and-software-sharing#loc-sharing-code and ensure that your code is shared in a way that follows best practice and facilitates reproducibility and reuse.

3. Thank you for stating in your Funding Statement:

“This work was supported by the National Natural Science Foundation of China under Grant No.62262044 and the Natural Science Foundation of Guangxi Province under Grant No.2023GXNSFAA026027.”

Please provide an amended statement that declares *all* the funding or sources of support (whether external or internal to your organization) received during this study, as detailed online in our guide for authors at http://journals.plos.org/plosone/s/submit-now.  Please also include the statement “There was no additional external funding received for this study.” in your updated Funding Statement.

Please include your amended Funding Statement within your cover letter. We will change the online submission form on your behalf.

4. Thank you for stating the following financial disclosure:

“This work was supported by the National Natural Science Foundation of China under Grant No.62262044 and the Natural Science Foundation of Guangxi Province under Grant No.2023GXNSFAA026027.”

Please state what role the funders took in the study.  If the funders had no role, please state: "The funders had no role in study design, data collection and analysis, decision to publish, or preparation of the manuscript."

If this statement is not correct you must amend it as needed.

Please include this amended Role of Funder statement in your cover letter; we will change the online submission form on your behalf.

5. Please note that your Data Availability Statement is currently missing [the repository name and/or the DOI/accession number of each dataset OR a direct link to access each database]. If your manuscript is accepted for publication, you will be asked to provide these details on a very short timeline. We therefore suggest that you provide this information now, though we will not hold up the peer review process if you are unable.

6. Thank you for stating the following in the Acknowledgments Section of your manuscript:

“This work was supported by the National Natural Science Foundation of China under Grant No.62262044 and the Natural Science Foundation of Guangxi Province under Grant No.2023GXNSFAA026027.”

We note that you have provided additional information within the Acknowledgements Section that is not currently declared in your Funding Statement. Please note that funding information should not appear in the Acknowledgments section or other areas of your manuscript. We will only publish funding information present in the Funding Statement section of the online submission form.

Please remove any funding-related text from the manuscript and let us know how you would like to update your Funding Statement. Currently, your Funding Statement reads as follows:

“This work was supported by the National Natural Science Foundation of China under Grant No.62262044 and the Natural Science Foundation of Guangxi Province  under Grant No.2023GXNSFAA026027.”

Please include your amended statements within your cover letter; we will change the online submission form on your behalf.

7. If the reviewer comments include a recommendation to cite specific previously published works, please review and evaluate these publications to determine whether they are relevant and should be cited. There is no requirement to cite these works unless the editor has indicated otherwise.

[Note: HTML markup is below. Please do not edit.]

Reviewers' comments:

Reviewer's Responses to Questions

**Comments to the Author**

1. Is the manuscript technically sound, and do the data support the conclusions?

The manuscript must describe a technically sound piece of scientific research with data that supports the conclusions. Experiments must have been conducted rigorously, with appropriate controls, replication, and sample sizes. The conclusions must be drawn appropriately based on the data presented.

Reviewer #1: Partly

Reviewer #2: Partly

Reviewer #3: Yes

**********

2. Has the statistical analysis been performed appropriately and rigorously?

Reviewer #1: Yes

Reviewer #2: No

Reviewer #3: Yes

**********

3. Have the authors made all data underlying the findings in their manuscript fully available?

The PLOS Data policy requires authors to make all data underlying the findings described in their manuscript fully available without restriction, with rare exception (please refer to the Data Availability Statement in the manuscript PDF file). The data should be provided as part of the manuscript or its supporting information, or deposited to a public repository. For example, in addition to summary statistics, the data points behind means, medians and variance measures should be available. If there are restrictions on publicly sharing data—e.g. participant privacy or use of data from a third party—those must be specified.

Reviewer #1: No

Reviewer #2: No

Reviewer #3: Yes

**********

4. Is the manuscript presented in an intelligible fashion and written in standard English?

PLOS ONE does not copyedit accepted manuscripts, so the language in submitted articles must be clear, correct, and unambiguous. Any typographical or grammatical errors should be corrected at revision, so please note any specific errors here.

Reviewer #1: Yes

Reviewer #2: Yes

Reviewer #3: Yes

**********

5. Review Comments to the Author

Please use the space provided to explain your answers to the questions above. You may also include additional comments for the author, including concerns about dual publication, research ethics, or publication ethics. (Please upload your review as an attachment if it exceeds 20,000 characters)

Reviewer #1: The work of Lin et al. describes a new machine learning approach to predict drug-target interactions by coupling a global self-attention pooled graph neural network to learn meaningful features with a pre-trained transformer-based model to include semantic relationships. While the approach itself is interesting, the paper requires some major revision to be valuable for publication.

Unfortunately, the paper lacks the GitHub link for the dataset as well as references to the benchmarking datasets. Therefore, it is hard to evaluate their suitability for the approach. With regard to the benchmarking datasets, I would also expect a more detailed description of these datasets. What kind of data is in there and how were these datasets generated? Which limitations might be associated with their collection and how were positive and negative samples determined? Depending on the collection method, the benchmarking datasets might also include many false positive interactions. These limitations of the benchmarking datasets also needs to be discussed.

The authors compared their method on the Human dataset against ten methods while the C. elegans and BindingDB dataset was only compared against eight and six methods. What is the reason for the missing comparisons? In this sense, I would expect to include the missing comparisons or at least discuss why they are missing.

The authors compare themselves to methods that are published up to 2023, but state that their method is the new state of the art. Were there no further methods published between 2023 and 2025?

A critical evaluation of the approach itself and the applied benchmark dataset would be beneficial for the discussion. Are the mis-identified drug-target interactions similar between the different methods and do these sequences or drugs have something in common?

A reduced tone would be beneficial for the abstract. Of course machine learning can support the discovery of drug-interactions but they might only reduce laboratory efforts to check only the most promising interactions.

The authors report that their method learns more meaningful features of drug molecules by paying more attention to information features of certain important atomic nodes of the molecular structure. This raises the question for me whether there are common patterns in these molecules that are responsible for this effect. Similarly, the assignment of weights to nodes (line 315), including recommendations on how to assign these weights, requires a more detailed description, including the consequences.

All figure legends require a more detailed description. A title alone is not enough to enable the reader to understand what is depicted in the figure.

Figure 4 is blurred and needs to be replaced.

Avoid colloquial formulations e.g., and so on (line 119).

According to standard naming conventions, the names of organisms must be in italics (e.g. C. elegans).

Reviewer #2: The study proposes a new deep learning–based methodology, T-pGNN4DTI, for predicting drug–target interactions. The approach is evaluated on three benchmark datasets (C. elegans, Human, and BindingDB) and reportedly outperforms prior methods. However, several important issues must be addressed before the work can be considered technically sound and reproducible.

1. Insufficient explanation of the methodological novelty and integration

The manuscript introduces a global self-attention pooling module and several combined components (graph neural network, protein pre-training model, and feature fusion), yet the originality and integration of these elements remain unclear. Please clarify which parts of the framework are newly developed and which are adapted from prior studies. The authors should also explain why the global self-attention pooling mechanism was introduced, how it differs from existing pooling strategies, and how it affects downstream performance. A concise schematic or ablation focusing on this component’s contribution would improve clarity.

2. Missing dataset and unavailable code

The manuscript does not provide access to the datasets used in the study, nor does it include a GitHub or other code repository link. Data and code availability are essential for ensuring reproducibility and independent verification.

3. Lack of statistical rigor

The current results section lacks sufficient statistical validation. The manuscript reports single-run values for AUC, Precision, and Recall, but does not show whether these results are stable or statistically significant.

• No information is given on how many times each experiment was repeated.

• No averages, standard deviations, or confidence intervals are reported to indicate result variability.

• No statistical tests are conducted to verify that improvements over baselines are meaningful.

The authors should repeat experiments multiple times with different random seeds, report mean ± SD or confidence intervals, specify the number of samples used, and perform significance testing when comparing against other methods.

4. Hyperparameter configuration and potential overfitting risk

In most comparable studies, the number of training epochs does not exceed 500 and dropout rates typically range from 0.2 to 0.5 (e.g., GraphDTA, SAG-DTA, CPGL, TransformerCPI). In contrast, the present study trains for 1000 epochs with a very low dropout rate of 0.1, yet reports nearly perfect performance (AUC ≈ 0.998). This combination suggests a substantial risk of overfitting.

To ensure the reported performance is reliable and not a result of overfitting, the authors should provide learning curves showing training and validation loss across epochs, or at least describe the use of early-stopping criteria. Additionally, for fair comparison, baseline methods should be trained under similar or equivalent hyperparameter settings.

5.Language and formatting

In lines 283–294 and 299, the improvements are expressed in decimals (e.g., “0.015 higher”), whereas in lines 274–282 and 295–298 they are expressed in percentages (e.g., “0.52% to 78.82% higher”).

Overall recommendation

The manuscript requires substantial revision to clarify the methodological contributions, ensure data and code availability, and strengthen the statistical and experimental evidence supporting the conclusions.

Reviewer #3: The authors propose T-pGNN4DTI, an innovative drug-target interaction prediction method that combines global self-attention pooling graph neural networks with a Transformer-based protein pre-training model. This method effectively extracts more comprehensive and meaningful feature information by selectively focusing on key atomic nodes in drug molecules and capturing semantic relationships in long protein sequences. Experiments on multiple benchmark datasets demonstrate that T-pGNN4DTI outperforms existing DTI prediction methods, providing a new approach for interaction prediction in drug development. However, this article suffers from the following problems.

1. The T-pGNN4DTI model proposed in the paper is essentially a combination of existing global self-attention pooling technology and pre-trained protein language models, but the paper fails to adequately explain why this combination is particularly suitable for DTI prediction. It is suggested that the authors elaborate on why global self-attention pooling can effectively capture atoms critical to interactions in drug molecules and why pre-trained protein language models better represent protein binding site features, from the perspective of biochemical mechanisms of drug-target interactions. The authors could introduce specific drug-target interaction cases to analyze how the model identifies key interaction sites, thereby providing a stronger theoretical foundation for the method.

2. The description of the PTR pre-training model in the paper lacks sufficient detail and fails to explain why PTR was chosen over other widely used protein pre-training models. The authors are advised to supplement the complete technical details of the PTR model, including the scale and source of the pre-training corpus, model architecture parameters, and training strategies. Additionally, comparative experiments with different pre-trained protein models should be conducted to analyze performance differences and their causes in DTI prediction tasks. Visualization analyses could also be employed to demonstrate how PTR captures key regions in protein sequences related to drug binding, thereby validating its applicability in DTI tasks.

3. Although the authors emphasize that the global self-attention mechanism can identify key atomic nodes in molecular structures, the paper lacks detailed interpretability analysis to demonstrate this. There is no visualization of attention weight distributions, nor analysis of whether the atomic nodes focused on by the model correspond to actual drug-target binding sites. It is recommended that the authors add an interpretability analysis section to enhance the model's explainability and provide valuable insights for drug design.

4. The paper does not provide computational complexity analysis or comparisons of training and inference times for the model. In practical applications, model efficiency is as important as accuracy, especially in large-scale virtual screening scenarios. Furthermore, the lack of discussion regarding the model's parameter count makes it difficult to assess its resource requirements compared to existing methods. It is recommended that the authors supplement the paper with a comprehensive analysis of computational efficiency.

5. It is recommended that the authors introduce more advanced methods for drug association prediction in the introduction, not limited to DTI research, focusing on summarizing innovative methods such as "Multi-view contrastive learning for drug-drug interaction event prediction" and "LLM-DDI: Leveraging Large Language Models for Drug-Drug Interaction Prediction on Biomedical Knowledge Graph." This would highlight the cutting-edge nature and scientific value of the authors' proposed method.

**********

6. PLOS authors have the option to publish the peer review history of their article (what does this mean?). If published, this will include your full peer review and any attached files.

If you choose “no”, your identity will remain anonymous but your review may still be made public.

**Do you want your identity to be public for this peer review?**  For information about this choice, including consent withdrawal, please see our Privacy Policy.

Reviewer #1: No

Reviewer #2: No

Reviewer #3: No

**********

[NOTE: If reviewer comments were submitted as an attachment file, they will be attached to this email and accessible via the submission site. Please log into your account, locate the manuscript record, and check for the action link "View Attachments". If this link does not appear, there are no attachment files.]

To ensure your figures meet our technical requirements, please review our figure guidelines: https://journals.plos.org/plosone/s/figures

You may also use PLOS’s free figure tool, NAAS, to help you prepare publication quality figures: https://journals.plos.org/plosone/s/figures#loc-tools-for-figure-preparation.

NAAS will assess whether your figures meet our technical requirements by comparing each figure against our figure specifications.

# Author comment: Author response to Decision Letter 1

**DOI:** 10.1371/journal.pone.0352250.r002

13 Mar 2026

Dear reviewers�

Thank you very much for your comments and professional advice. We sincerely appreciate the editors and reviewers for their invaluable insights and suggestions, which were essential in improving our manuscript. We have carefully revised the manuscript according to the reviewers' comments and suggestions. The main changes are highlighted in RED and BLUE in the ANNOTATED VERSION. Following is our point-by-point response to the reviewers' comments.

Best regards,

Yuzhong Peng

On behalf of all co-authors

To reviewer 1:

Comment 1: Unfortunately, the paper lacks the GitHub link for the dataset as well as references to the benchmarking datasets. Therefore, it is hard to evaluate their suitability for the approach. With regard to the benchmarking datasets, I would also expect a more detailed description of these datasets. What kind of data is in there and how were these datasets generated? Which limitations might be associated with their collection and how were positive and negative samples determined? Depending on the collection method, the benchmarking datasets might also include many false positive interactions. These limitations of the benchmarking datasets also needs to be discussed.

Response 1: Thank you for your valuable suggestions. To make the changes easier to identify where necessary, I have decomposed Comment 1 into the sub-comments numbered as follows:

1.1 The paper lacks the GitHub link for the dataset as well as references to the benchmarking datasets.

1.2 I would also expect a more detailed description of these datasets, including the following issues: What kind of data is in there and how were these datasets generated? Which limitations might be associated with their collection and how were positive and negative samples determined?

1.3 Depending on the collection method, the benchmarking datasets might also include many false positive interactions. These limitations of the benchmarking datasets also need to be discussed.

According to your suggestions, we have tried our best to revise our manuscript to meet your suggestions, as follows:

Response 1.1: We have added a Section “Data and code availability” to give source for data and code availability.

Response 1.2: We have added a more detailed description of the benchmarking datasets in Section “Benchmark datasets” to meet your suggestions. The added detailed description mainly includes: “The dataset employed in this study follows TransformerCPI~\cite{chen2020transformercpi}, drawing from three publicly available benchmark datasets widely recognized in the DTA prediction field in recent years\cite{Zhou2015Improving, Zhang2023A}, including the BindingDB, Human, and C.elegans datasets. Positive samples for the Human and C.elegans datasets were derived from positive drug-protein interaction (CPI) pairs in DrugBank 4.1 and Matador~\cite{chen2020transformercpi}, with high-confidence negative samples generated through the negative CPI screening framework ~\cite{Zhou2015Improving}. A more detailed introduction to the three benchmark datasets is given below.

BindingDB is a publicly accessible large-scale database measuring binding affinities, primarily focusing on interactions between proteins considered drug targets and drug-like small molecules~\cite{BindingDB}. The BindingDB dataset used in this work was obtained from TransformerCPI~\cite{chen2020transformercpi}, containing 39747 positive interactions (involving 1696 protein targets and 53253 small molecules) and 31218 negative samples. The C.elegans dataset contains extensive information on gene, protein, and compound interactions in the nematode Caenorhabditis elegans, including 4000 positive interactions between 1434 unique compounds and 2,504 unique proteins specific to the C.elegans species. The Human dataset contains 852 drugs and 1052 proteins, covering 3369 interaction pairs.”

Response 1.3: We have added a discussion of the benchmarking datasets’ limitations in the “Discussion” section to address your suggestions. The added detailed description mainly includes:

“However, the benchmarking datasets are not ideal due to limitations in data collection methods and experimental conditions. For example, most drugs in the Human and BindingDB dataset only occur in one class, potentially introducing bias into feature learning. The relatively small sample size of compound-protein interactions (CPIs) in Human and C.elegans may limit the training of complex deep learning models. Furthermore, negative samples are generated by algorithms that may introduce undetectable noise~\cite{Zhou2015Improving}, necessitating biological experimental corrections by the scientific community.”

Comment 2: The authors compared their method on the Human dataset against ten methods while the C. elegans and BindingDB dataset was only compared against eight and six methods. What is the reason for the missing comparisons? In this sense, I would expect to include the missing comparisons or at least discuss why they are missing.

Response 2: Thank you for your comment. The reason for the missing comparisons is as follows: the comparative data in the manuscript were partially reproduced by the authors (including GCN, FusionDTI, and two variant models of SAG) and partially cited from relevant literatures. However, some original papers did not cover all benchmark datasets or had differences in experimental settings and evaluation metrics, making fair comparisons impossible and thus not included in our manuscript. For instance, the original papers for DrugVQA and AMMVF-DTI present results for the Human dataset but omit results for the C. elegans and BindingDB datasets. Consequently, these two methods are not compared in the C. elegans and BindingDB dataset experiments, resulting in a difference in the number of methods compared across datasets. These resulted in an inconsistent number of comparison methods across different datasets.

Comment 3: The authors compare themselves to methods that are published up to 2023, but state that their method is the new state of the art. Were there no further methods published between 2023 and 2025?

Response 3: Thank you for your valuable comment. We have added 5 state-of-the-art methods in the revised manuscript, including two (IMAEN and MGNDTI) published in 2024 , two (CF-DTI and FusionDTI) published in 2025, and one (TopoPharmDTI) published in 2026. As demonstrated in Table 3~5. All the comparative SOTA models underperformed T-pGNN4DTI. More detailed can be seen in Sections “Baseline models” and “Comparisons results” in revised manuscript. This demonstrates that T-pGNN4DTI is an effective method for improving DTI prediction.

Comment 4: A critical evaluation of the approach itself and the applied benchmark dataset would be beneficial for the discussion. Are the mis-identified drug-target interactions similar between the different methods and do these sequences or drugs have something in common?

Response 4: Thank you for your insightful comment. Following your suggestion, we systematically analyzed the misclassified drug-target interactions in the GCN and FusionDTI models reproduced in this paper and our proposed model. We found that (1) significant overlap exists among the misclassified drug-target interaction samples across the three models. Many samples misclassified by GCN and FusionDTI were also misclassified by our proposed model. This may be because some key biological features of these samples in DTIs can not be fully exploited by these deep learning models; (2) most misclassified samples within each model did not exhibit strong similarities in sequence or drug structure. Moving forward, we will employ more advanced methods and techniques to investigate whether more subtle common features exist, and we will incorporate your suggestions into our future research endeavors.

Comment 5: A reduced tone would be beneficial for the abstract. Of course machine learning can support the discovery of drug-interactions but they might only reduce laboratory efforts to check only the most promising interactions.

Response 5: Thank you very much for your insightful suggestions. We have revised the phrasing of the third and final sentences in the abstract according to your suggestions. i.g., “Machine learning-based methods can rapidly predict DTI using only computer algorithmic models, allowing researchers to validate only the most promising interactions through biochemical experiments. This holds promise for effectively addressing the current challenges of lengthy development cycles and high costs in new drug development.”,“It provides a new way of thinking to help solve the DTI-related problems.”

Comment 6: The authors report that their method learns more meaningful features of drug molecules by paying more attention to information features of certain important atomic nodes of the molecular structure. This raises the question for me whether there are common patterns in these molecules that are responsible for this effect. Similarly, the assignment of weights to nodes (line 315), including recommendations on how to assign these weights, requires a more detailed description, including the consequences.

Response 6: Thank you for your valuable suggestions. To make the changes easier to identify where necessary, I have decomposed Comment 6 into the sub-comments numbered as follows:

6.1 This raises the question for me whether there are common patterns in these molecules that are responsible for this effect.

6.2 Similarly, the assignment of weights to nodes (line 315), including recommendations on how to assign these weights, requires a more detailed description, including the consequences.

According to your suggestions, we have tried our best to revise our manuscript to meet your suggestions, as follows:

Response 6.1: Yes, common patterns among molecules in the training set are captured by the attention mechanism algorithm, thereby inducing this effect.

Response 6.2: We have added detailed explanations on how these weights are assigned in the revision, such as lines 264-278 of the “Drug Feature Extraction” section. Specific examples are provided in the “T-pGNN4DTI Case Studies and Interpretability” section, where you can further understand the weight assignment method and its effects (as shown in Figure 6).

Comment 7: All figure legends require a more detailed description. A title alone is not enough to enable the reader to understand what is depicted in the figure.

Response 7: Thank you very much for your valuable suggestions. We have supplemented each figure legend with a more detailed description.

Comment 8: Figure 4 is blurred and needs to be replaced.

Response 8: Thank you very much for your valuable suggestions. We have redrawn a clearer version of Figure 4.

Comment 9: Avoid colloquial formulations, e.g., and so on (line 119).

Response 9: Thank you very much for your valuable suggestions. We have re-optimized the language expression of the manuscript to make it more standardized, smoother, and logically more rigorous in the revision. e.g., lines 90-92, 139-140,142-144, 136-137, 147-149, 175-176, 218-223, 235-236,238-243, 247-252, 263-265,293,303,479-480,502-503 and so on.

Comment 10: According to standard naming conventions, the names of organisms must be in italics (e.g., C. elegans).

Response 10: Thank you very much for your insightful suggestions. We have adopted your suggestion and italicized all organism names (including C. elegans and humans) in accordance with standard naming conventions.

To reviewer 2:

Comment 1: Insufficient explanation of the methodological novelty and integration

The manuscript introduces a global self-attention pooling module and several combined components (graph neural network, protein pre-training model, and feature fusion), yet the originality and integration of these elements remain unclear. Please clarify which parts of the framework are newly developed and which are adapted from prior studies. The authors should also explain why the global self-attention pooling mechanism was introduced, how it differs from existing pooling strategies, and how it affects downstream performance. A concise schematic or ablation focusing on this component’s contribution would improve clarity.

Response 1: Thanks for your valuable suggestions. The reviewers commented on the issue “Insufficient explanation of the methodological novelty and integration”. To make the changes easier to identify where necessary, I have decomposed Comment 1 into the sub-comments numbered as follows:

1.1 The originality and integration of these elements remain unclear. Please clarify which parts of the framework are newly developed and which are adapted from prior studies.

1.2 The authors should also explain why the global self-attention pooling mechanism was introduced and how it differs from existing pooling strategies.

1.3 How it affects downstream performance.

1.4 A concise schematic or ablation focusing on this component’s contribution would improve clarity.

Response 1.1: Thank you for pointing this out. Our T-pGNN4DTI is not a simple stacking of existing components. Instead, our T-pGNN4DTI proposes an integrative innovation strategy to tackle the challenge of drug-target interaction prediction. This strategy involves the synergistic and complementary integration of three major components, including protein feature learning, compound feature learning, and feature fusion with pattern recognition.

It integrates three complementary and synergistic components (including a protein pre-training model, global attention pooling graph convolutional neural networks, and concatenation + MLP) and end-to-end training. This high-performance drug-target interaction prediction method embodies an innovative tripartite integration mechanism of “generalization-focus-efficiency”. Compared to existing single-technique approaches (e.g., relying solely on pre-trained models, recurrent neural networks, or graph neural networks) or fusion techniques (e.g., LSTM+CNN, LSTM+GAT, protein pre-trained models + compound pre-trained models, or other complex fusion architectures), our methodology's core innovation lies in the complementary synergy of its components and end-to-end optimization. Together, these overcome bottlenecks of data sparsity, feature fragmentation, and computational efficiency, achieving DTI prediction performance that surpasses existing methods. See the SOTA comparison results (Tables 3–5) and ablation study results (Table 6) in the “Experimental Results” section of the revision.

Specifically, existing protein sequence learning models (e.g., TransformerCPI) heavily rely on labeled sample data. Our T-pGNN4DTI leverages pre-trained protein models to learn evolutionary conservation, sequence patterns, and structural priors, generating robust, generalized protein features that significantly reduce dependence on labeled data and address data sparsity. Existing standard graph neural networks and GATs (e.g., GraphDTA) employ global average pooling, overlooking local interaction details between compounds and proteins. Our T-pGNN4DTI uses globally attentive pooling graph networks to learn compound features. By dynamically weighting node importance (e.g., atoms, functional groups, and substructures), it enhances focus on and capture of critical compound substructures during drug-target interactions, improving interpretability. It demonstrates significant advantages over traditional graph neural networks in noise robustness, structural fault tolerance, and data missing compensation. Existing feature fusion methods often involve computationally complex operations (e.g., DMFF-DTA), resulting in high computational overhead that hinders scalability. Our T-pGNN4DTI employs feature concatenation and MLP to integrate and align learned protein and compound features, ensuring lossless transmission of sequence and structural information. This effectively combines local features of drugs and targets, successfully mining key biological characteristics in drug-target interactions and enabling lightweight, efficient drug-target interaction prediction.

In summary, although both the PTR and self-attention pooling neural networks in our methodology draw inspiration from existing methods, the core components of

Attachment Submitted filename: Response to Reviewers.docx

# Peer review: Decision Letter 1

**DOI:** 10.1371/journal.pone.0352250.r003

**Authors:** Lun Hu

22 Apr 2026

PONE-D-25-56836R1

T-pGNN4DTI: Towards Better Drug-target Interactions Prediction Using Global Self-attentive Pooled Graph Convolutional Networks and Protein Pre-training Models

PLOS One

Dear Dr. Peng,

Thank you for submitting your manuscript to PLOS ONE. After careful consideration, we feel that it has merit but does not fully meet PLOS ONE’s publication criteria as it currently stands. Therefore, we invite you to submit a revised version of the manuscript that addresses the points raised during the review process.

Please submit your revised manuscript by Jun 06 2026 11:59PM. If you will need more time than this to complete your revisions, please reply to this message or contact the journal office at plosone@plos.org. When you're ready to submit your revision, log on to https://www.editorialmanager.com/pone/ and select the 'Submissions Needing Revision' folder to locate your manuscript file.

Please include the following items when submitting your revised manuscript:

- A letter that responds to each point raised by the academic editor and reviewer(s). You should upload this letter as a separate file labeled 'Response to Reviewers'.
- A marked-up copy of your manuscript that highlights changes made to the original version. You should upload this as a separate file labeled 'Revised Manuscript with Track Changes'.
- An unmarked version of your revised paper without tracked changes. You should upload this as a separate file labeled 'Manuscript'.

If you would like to make changes to your financial disclosure, please include your updated statement in your cover letter. Guidelines for resubmitting your figure files are available below the reviewer comments at the end of this letter.

If applicable, we recommend that you deposit your laboratory protocols in protocols.io to enhance the reproducibility of your results. Protocols.io assigns your protocol its own identifier (DOI) so that it can be cited independently in the future. For instructions see: https://journals.plos.org/plosone/s/submission-guidelines#loc-laboratory-protocols. Additionally, PLOS ONE offers an option for publishing peer-reviewed Lab Protocol articles, which describe protocols hosted on protocols.io. Read more information on sharing protocols at https://plos.org/protocols?utm_medium=editorial-email&utm_source=authorletters&utm_campaign=protocols.

As the corresponding author, your ORCID iD is verified in the submission system and will appear in the published article. PLOS supports the use of ORCID, and we encourage all coauthors to register for an ORCID iD and use it as well. Please encourage your coauthors to verify their ORCID iD within the submission system before final acceptance, as unverified ORCID iDs will not appear in the published article. *Only*  the individual author can complete the verification step; PLOS staff *cannot*  verify ORCID iDs on behalf of authors.

We look forward to receiving your revised manuscript.

Kind regards,

Lun Hu

Academic Editor

PLOS One

Journal Requirements:

If the reviewer comments include a recommendation to cite specific previously published works, please review and evaluate these publications to determine whether they are relevant and should be cited. There is no requirement to cite these works unless the editor has indicated otherwise.

Please review your reference list to ensure that it is complete and correct. If you have cited papers that have been retracted, please include the rationale for doing so in the manuscript text, or remove these references and replace them with relevant current references. Any changes to the reference list should be mentioned in the rebuttal letter that accompanies your revised manuscript. If you need to cite a retracted article, indicate the article’s retracted status in the References list and also include a citation and full reference for the retraction notice.

[Note: HTML markup is below. Please do not edit.]

Reviewers' comments:

Reviewer's Responses to Questions

**Comments to the Author**

1. If the authors have adequately addressed your comments raised in a previous round of review and you feel that this manuscript is now acceptable for publication, you may indicate that here to bypass the “Comments to the Author” section, enter your conflict of interest statement in the “Confidential to Editor” section, and submit your "Accept" recommendation.

Reviewer #2: (No Response)

Reviewer #3: All comments have been addressed

**********

2. Is the manuscript technically sound, and do the data support the conclusions?

The manuscript must describe a technically sound piece of scientific research with data that supports the conclusions. Experiments must have been conducted rigorously, with appropriate controls, replication, and sample sizes. The conclusions must be drawn appropriately based on the data presented.

Reviewer #2: Yes

Reviewer #3: Yes

**********

3. Has the statistical analysis been performed appropriately and rigorously?

Reviewer #2: Yes

Reviewer #3: Yes

**********

4. Have the authors made all data underlying the findings in their manuscript fully available?

The PLOS Data policy requires authors to make all data underlying the findings described in their manuscript fully available without restriction, with rare exception (please refer to the Data Availability Statement in the manuscript PDF file). The data should be provided as part of the manuscript or its supporting information, or deposited to a public repository. For example, in addition to summary statistics, the data points behind means, medians and variance measures should be available. If there are restrictions on publicly sharing data—e.g. participant privacy or use of data from a third party—those must be specified.

Reviewer #2: No

Reviewer #3: Yes

**********

5. Is the manuscript presented in an intelligible fashion and written in standard English?

PLOS ONE does not copyedit accepted manuscripts, so the language in submitted articles must be clear, correct, and unambiguous. Any typographical or grammatical errors should be corrected at revision, so please note any specific errors here.

Reviewer #2: Yes

Reviewer #3: Yes

**********

6. Review Comments to the Author

Please use the space provided to explain your answers to the questions above. You may also include additional comments for the author, including concerns about dual publication, research ethics, or publication ethics. (Please upload your review as an attachment if it exceeds 20,000 characters)

Reviewer #2: Thank you for the revision. The manuscript has improved, but I still have two remaining concerns that should be addressed before publication.

First, I am still concerned that the current revision does not fully rule out the possibility of over-optimistic model performance. In particular, I could not find the corresponding results or figure that would help evaluate the relationship between prediction accuracy and false negatives more clearly. Without such evidence, the reported performance still appears somewhat too good to be fully convincing. I therefore encourage the authors to provide the relevant analysis, figure, or discussion to better demonstrate that the model is not overfitted and that the predictive performance is robust. Providing additional validation (e.g., cross-validation results, independent test sets, or robustness analyses) would further strengthen confidence in the reported performance.

Second, at least one of the links provided in the manuscript appears not to be accessible. In particular, the GitHub link around line 538 could not be opened when I checked it. Please verify that all links are correct, active, and publicly accessible, as this is important for reproducibility and for allowing readers and reviewers to examine the associated code or resources.

Overall, I believe the manuscript is improved, but these remaining issues should still be addressed.

Reviewer #3: (No Response)

**********

7. PLOS authors have the option to publish the peer review history of their article (what does this mean?). If published, this will include your full peer review and any attached files.

If you choose “no”, your identity will remain anonymous but your review may still be made public.

**Do you want your identity to be public for this peer review?**  For information about this choice, including consent withdrawal, please see our Privacy Policy.

Reviewer #2: No

Reviewer #3: No

**********

[NOTE: If reviewer comments were submitted as an attachment file, they will be attached to this email and accessible via the submission site. Please log into your account, locate the manuscript record, and check for the action link "View Attachments". If this link does not appear, there are no attachment files.]

To ensure your figures meet our technical requirements, please review our figure guidelines: https://journals.plos.org/plosone/s/figures

You may also use PLOS’s free figure tool, NAAS, to help you prepare publication quality figures: https://journals.plos.org/plosone/s/figures#loc-tools-for-figure-preparation.

NAAS will assess whether your figures meet our technical requirements by comparing each figure against our figure specifications.

# Author comment: Author response to Decision Letter 2

**DOI:** 10.1371/journal.pone.0352250.r004

3 Jun 2026

We particularly appreciate the reviewer for his/her insights and comments, which were essential in improving our manuscript entitled “T-pGNN4DTI: Towards Better Drug-target Interactions Prediction Using Global Self-attentive Pooled Graph Convolutional Networks and Protein Pre-training Models” (Manuscript Number: PONE-D-25-56836). We have carefully revised the manuscript according to the reviewers' comments and suggestions. The main changes are highlighted in RED in the ANNOTATED VERSION. The file “Response to Reviewers20260602.pdf” detailed our point-by-point response to the reviewers' comments.

We hope that the corrections will meet with approval. If you have any questions, please don’t hesitate to contact me at the address below.

Thank you and best regards.

Yours sincerely,

Yuzhong Peng (Corresponding author)

E-mail: jedison@163.com

Attachment Submitted filename: Response to Reviewers20260602.pdf

# Peer review: Decision Letter 2

**DOI:** 10.1371/journal.pone.0352250.r005

**Authors:** Lun Hu

8 Jun 2026

T-pGNN4DTI: Towards Better Drug-target Interactions Prediction Using Global Self-attentive Pooled Graph Convolutional Networks and Protein Pre-training Models

PONE-D-25-56836R2

Dear Dr. Peng,

We’re pleased to inform you that your manuscript has been judged scientifically suitable for publication and will be formally accepted for publication once it meets all outstanding technical requirements.

Within one week, you’ll receive an e-mail detailing the required amendments. When these have been addressed, you’ll receive a formal acceptance letter and your manuscript will be scheduled for publication.

An invoice will be generated when your article is formally accepted. Please note, if your institution has a publishing partnership with PLOS and your article meets the relevant criteria, all or part of your publication costs will be covered. Please make sure your user information is up-to-date by logging into Editorial Manager at Editorial Manager® and clicking the ‘Update My Information' link at the top of the page. For questions related to billing, please contact billing support.

If your institution or institutions have a press office, please notify them about your upcoming paper to help maximize its impact. If they’ll be preparing press materials, please inform our press team as soon as possible -- no later than 48 hours after receiving the formal acceptance. Your manuscript will remain under strict press embargo until 2 pm Eastern Time on the date of publication. For more information, please contact onepress@plos.org.

Kind regards,

Lun Hu

Academic Editor

PLOS One

Additional Editor Comments (optional):

All reviewers were satisified with the changes made in this revised version of the manuscript.

Reviewers' comments:

Reviewer's Responses to Questions

**Comments to the Author**

1. If the authors have adequately addressed your comments raised in a previous round of review and you feel that this manuscript is now acceptable for publication, you may indicate that here to bypass the “Comments to the Author” section, enter your conflict of interest statement in the “Confidential to Editor” section, and submit your "Accept" recommendation.

Reviewer #2: All comments have been addressed

**********

2. Is the manuscript technically sound, and do the data support the conclusions?

The manuscript must describe a technically sound piece of scientific research with data that supports the conclusions. Experiments must have been conducted rigorously, with appropriate controls, replication, and sample sizes. The conclusions must be drawn appropriately based on the data presented.

Reviewer #2: Yes

**********

3. Has the statistical analysis been performed appropriately and rigorously?

Reviewer #2: Yes

**********

4. Have the authors made all data underlying the findings in their manuscript fully available?

The PLOS Data policy requires authors to make all data underlying the findings described in their manuscript fully available without restriction, with rare exception (please refer to the Data Availability Statement in the manuscript PDF file). The data should be provided as part of the manuscript or its supporting information, or deposited to a public repository. For example, in addition to summary statistics, the data points behind means, medians and variance measures should be available. If there are restrictions on publicly sharing data—e.g. participant privacy or use of data from a third party—those must be specified.

Reviewer #2: Yes

**********

5. Is the manuscript presented in an intelligible fashion and written in standard English?

PLOS ONE does not copyedit accepted manuscripts, so the language in submitted articles must be clear, correct, and unambiguous. Any typographical or grammatical errors should be corrected at revision, so please note any specific errors here.

Reviewer #2: Yes

**********

6. Review Comments to the Author

Please use the space provided to explain your answers to the questions above. You may also include additional comments for the author, including concerns about dual publication, research ethics, or publication ethics. (Please upload your review as an attachment if it exceeds 20,000 characters)

Reviewer #2: I thank the authors for carefully addressing the reviewer comments and substantially improving the manuscript.

The revised manuscript includes additional statistical analyses, ablation studies, interpretability analyses, and discussion of limitations. The methodology is clearly described, the experimental evaluation is comprehensive, and the conclusions are supported by the presented results.

I have no further major concerns and believe that the manuscript is suitable for publication in PLOS ONE in its current form.

**********

7. PLOS authors have the option to publish the peer review history of their article (what does this mean?). If published, this will include your full peer review and any attached files.

If you choose “no”, your identity will remain anonymous but your review may still be made public.

**Do you want your identity to be public for this peer review?**  For information about this choice, including consent withdrawal, please see our Privacy Policy.

Reviewer #2: No

**********

# Editor report: Acceptance letter

**DOI:** 10.1371/journal.pone.0352250.r006

**Authors:** Lun Hu

PONE-D-25-56836R2

PLOS One

Dear Dr. Peng,

I'm pleased to inform you that your manuscript has been deemed suitable for publication in PLOS One. Congratulations! Your manuscript is now being handed over to our production team.

At this stage, our production department will prepare your paper for publication. This includes ensuring the following:

* All references, tables, and figures are properly cited

* All relevant supporting information is included in the manuscript submission,

* There are no issues that prevent the paper from being properly typeset

You will receive further instructions from the production team, including instructions on how to review your proof when it is ready. Please keep in mind that we are working through a large volume of accepted articles, so please give us a few days to review your paper and let you know the next and final steps.

Lastly, if your institution or institutions have a press office, please let them know about your upcoming paper now to help maximize its impact. If they'll be preparing press materials, please inform our press team within the next 48 hours. Your manuscript will remain under strict press embargo until 2 pm Eastern Time on the date of publication. For more information, please contact onepress@plos.org.

You will receive an invoice from PLOS for your publication fee after your manuscript has reached the completed accept phase. If you receive an email requesting payment before acceptance or for any other service, this may be a phishing scheme. Learn how to identify phishing emails and protect your accounts at https://explore.plos.org/phishing.

If we can help with anything else, please email us at customercare@plos.org.

Thank you for submitting your work to PLOS ONE and supporting open access.

Kind regards,

PLOS ONE Editorial Office Staff

on behalf of

Dr. Lun Hu

Academic Editor

PLOS One
